# The Effects of Vegetarian and Vegan Diet during Pregnancy on the Health of Mothers and Offspring

**DOI:** 10.3390/nu11030557

**Published:** 2019-03-06

**Authors:** Giorgia Sebastiani, Ana Herranz Barbero, Cristina Borrás-Novell, Miguel Alsina Casanova, Victoria Aldecoa-Bilbao, Vicente Andreu-Fernández, Mireia Pascual Tutusaus, Silvia Ferrero Martínez, María Dolores Gómez Roig, Oscar García-Algar

**Affiliations:** 1 Neonatology Unit, Hospital Clinic-Maternitat, ICGON, BCNatal, 08028 Barcelona, Spain; HERRANZ@clinic.cat (A.H.B.); CBORRASN@clinic.cat (C.B.-N.); MMALSINA@clinic.cat (M.A.C.); VALDECOA@clinic.cat (V.A.-B.); mireiapascualt@gmail.com (M.P.T.); OGARCIAA@clinic.cat (O.G.-A.); 2 Grup de Recerca Infancia i Entorn (GRIE), Institut d’investigacions Biomèdiques August Pi i Sunyer (IDIBAPS), 08028 Barcelona, Spain; VIANDREU@clinic.cat; 3 BCNatal | Barcelona Center for Maternal Fetal and Neonatal Medicine Hospital Sant Joan de Déu, 08028 Barcelona, Spain; sferrero@sjdhospitalbarcelona.org (S.F.M.); lgomezroig@sjdhospitalbarcelona.org (M.D.G.R.)

**Keywords:** vegetarian diets, vegan diets, plant-based diets, nutrition, pregnancy, breastfeeding, human milk, micronutrients, fetal development

## Abstract

Vegetarian and vegan diets have increased worldwide in the last decades, according to the knowledge that they might prevent coronary heart disease, cancer, and type 2 diabetes. Althought plant-based diets are at risk of nutritional deficiencies such as proteins, iron, vitamin D, calcium, iodine, omega-3, and vitamin B12, the available evidence shows that well planned vegetarian and vegan diets may be considered safe during pregnancy and lactation, but they require a strong awareness for a balanced intake of key nutrients. A review of the scientific literature in this field was performed, focusing specifically on observational studies in humans, in order to investigate protective effects elicited by maternal diets enriched in plant-derived foods and possible unfavorable outcomes related to micronutrients deficiencies and their impact on fetal development. A design of pregestational nutrition intervention is required in order to avoid maternal undernutrition and consequent impaired fetal growth.

## 1. Introduction

Balanced maternal nutrition during pregnancy is imperative for the mother’s health status and, consequently, for offspring, and is crucial to maintain an adequate environment for optimal fetal development. According to the theory of “early life programming” environmental factors and lifestyle during pregnancy determine the risk of developing chronic diseases later in life and also influence lifelong health in offspring [1]. Pregnancy requires an increased intake of macro and micronutrients and balanced diet. For that, it offers a critical window of opportunity to acquire dietetic habits that are beneficial for fetal healthy [2].

Pre-pregnancy and pregnancy adherence to food-safety recommendations, according to the updated Dietary Guidelines for American and Mediterranean Diet, should avoid inadequate levels of key nutrients and micronutrients (proteins, iron, folic acid, vitamin D, calcium, iodine, omega-3, and vitamin B12) that may predispose the offspring to chronic condition later in life such as obesity, diabetes, cardiovascular disease, and neurodevelopmental delays [3]. 

The percentage of vegetarians and vegans in the general population has increased over the last years partly due to evidence that vegetarianism is linked to improved health. Thus, cohort data have shown that low-fat diets enriched with fruit, vegetables, and fiber can lead to a reduction of risk factors for coronary heart diseases, a better lipid profile [4], lower body mass index (BMI) [5], and lower blood pressure [6]. In addition vegetarian diets appear to prevent cancer and type 2 diabetes [7,8]. Nevertheless, some data suggest that vegetarians and vegans may be at greater risk of increased plasma homocysteine levels, an arising risk factor for cardiovascular disease [9,10], and of low bone mineral density, which predisposes to osteoporosis [11]. Plant-based diets are reported to contain less saturated fatty acids, animal protein and cholesterol, and more folate, fibre, antioxidants, phytochemicals, and carotenoids. However, plant-based diets have a low content of essential micronutrients such as iron, zinc, vitamin B 12, vitamin D, omega-3 (*n*-3) fatty acids, calcium, and iodine. Consequently, the risk of adverse effects due to micronutrient deficiencies that lead to the risk of malnutrition should not be underestimated [12].

The reasons for choosing a vegetarian or vegan lifestyle are variable and range from evidence-based health consciousness to environmental concerns, socioeconomic considerations, ethical grounds, or spiritual/religious beliefs. Medical reasons also exist in some occasions; for instance, women of childbearing age affected by chronic kidney disease (CKD) may be conditioned to the choice of low-protein vegetarian diet [13]. According to the American Dietetic Association, well planned vegetarian diets are safe for all age groups and in all physiological conditions, including childhood, adolescence, pregnancy, and lactation [12,14]. On the contrary, the German Nutrition Society does not recommend vegetarian or vegan diets during pregnancy, lactation, and childhood, due to the inadequate supply of essential nutrients [15].

Vegetarian diets typically comprise of plant foods such as grains, legumes, nuts, seeds, vegetables, and fruit, and exclude all kinds of animal food including meat (pork, beef, mutton, lamb, poultry, game, and fowl), meat products (sausages, salami, and pâté), fish, mollusks, and crustaceans. Vegetarian diets usually include dairy products like eggs and honey. Accordingly, there are two main directions:

(1) Lacto-ovo-vegetarianism (LOV). This excludes meat but includes dairy products, eggs, and honey, together with a wide variety of plant foods. Subcategories are lacto-vegetarianism (LV), which excludes eggs, and ovo-vegetarianism (OV), which excludes dairy products.
(2) Veganism (VEG), which excludes meat, dairy products, eggs, and honey, but includes a wide variety of plant foods [16].

However, some people adhere to other plant-based diets that limit the foods consumed:

- Raw food diet: consisting exclusively of vegetables, including sprouted cereals and pulses, fresh and dried fruits, and seeds, as well as milk and eggs, all of which are mainly eaten raw.
- Fruit diet: consisting exclusively of fresh and dried fruits, seeds, and some vegetables.
- Macrobiotic diet: the strictly vegetarian version of this diet consists of cereals, pulses, vegetables, seaweed, and soy products; while dairy products, eggs, and some vegetables are avoided. Fish is consumed by people who adhere to a macrobiotic diet.

Despite the concrete definition of such categories there is a wide variety of dietary pattern. Vegetarians can be divided into other subgroups: semivegetarians, who are defined as consuming red meat and poultry once per month or more and all meats—including fish—once per month or more, but no more than once per week; pesco-vegetarians, who consume fish once per month or more but all other meats less than once per month; lacto-vegetarians, who consume eggs and dairy once per month or more, but fish and other meats less tan once per month; and lastly, vegans or strict vegetarians are defined as those who do not consume eggs, dairy, or fish [16].

In Europe and North America vegetarians are LOV [17], while Asian Indian vegetarians are mostly lacto-vegetarians [18]. Moreover, it has been described that Chinese vegetarians consume considerably smaller amount of dairy products than Western vegetarians [19].

In 2006, approximately 2.3% of the US adult population (4.9 million people) strictly followed a vegetarian diet, asserting that they never ate meat, fish, or poultry. In 2012 the percentage increased to 5%. Approximately 1.4–2% of the US adult population is vegan [20]. The percentage of young people who are vegetarian is still higher (6–11%), with similar levels of vegetarian teenagers reported in both the United Kingdom and Australia [21]. On the basis of dietary recall data from the 1999–2004 National Health and Nutrition Examination Survey (NHANES), 7.5% of American women were reported adhering to a vegetarian diet [22]. The distribution of women of childbearing age who follow a vegetarian diet is different between developed and developing countries. In developing countries, the prevalence of vegetarian diet may increase due to poverty and economic reasons. In India, 20–30% of the total population is considered to be vegetarian for religious reasons, but normally they do not eat meat because of economic causes. In developed countries, vegetarians comprehend more women than men, and tend to be of higher educational or socioeconomic status, with low probabilities to plan children, and generally they are under 40 years of age. There are also variations between ethnic groups. In the United Kingdom, people of non-White ethnic origin are more likely to indicate themselves as vegetarian than white (15% vs. 6% of white respondents) [23].

Despite the assessment of the importance of a healthy diet in pregnancy, data have demonstrated that women don’t change diet during pregnancy, so optimal preconception dietary pattern is determinant for a healthy pregnancy [24]. In addition, the period of lactation is extremely important for growing patterns of infants and the effectiveness of breastfeeding depends on maternal nutritional status. The lack of macro- and micronutrient intake during lactation may lead to the reduction of micronutrients and energy content in breast milk that could lead to severe illness in breastfed infant [25].

The aim of this narrative review was to analyze the existing studies in humans focused on the effects of vegetarian (LOV) and vegan (VEG) diets during pregnancy on maternal outcomes and nutritional status and on fetal healthiness and complications, valuing the risks and benefits of such nutritional choice. Moreover our aim was to study the period of lactation and breast milk composition of vegetarian and vegan mothers and if the breastfeeding is safe for optimal child growth, because there are no specific clinical guidelines about lactation for vegetarian mothers. The publications reviewed in this paper mainly concern plant-based diets as LOV and VEG. Consequently, the results mainly pertain to these diets, which are generally defined as “vegetarian”. We did not assess studies about raw food, fruit, and macrobiotic diets. A research for English written articles was performed using MEDLINE/PubMed/Cochrane databases (since 2000 up to 31 December 2018). The research was based on the combination of the following keywords: vegetarian diet, vegan diet, plant-based nutrition, pregnancy outcomes, fetal development, vegetarian/vegan, and breastfeeding/human milk, combined with words related to nutritional status and to the nutrients of interest (protein, vitamin B12, folate, calcium, iron, zinc, iodine, and *n*-3 fatty acids). Studies were identified and examined for methodology and key results. Researches about preconception period were also analyzed. Review team members screened titles and abstracts and selected articles that seemed pertinent to the topics, excluding those not in English or not concerned with humans. The full papers were read to select potentially eligible articles and to assess the scientific merit and relevance of each paper valued. The manuscripts were also analyzed according with the type of study (case–control, review, longitudinal cohort, and cross-sectional), type of diets, number of cases, and possible bias. We performed a narrative review because we expected high heterogeneity of results and lack of randomised trials in pregnant vegetarians in the literature. We included American Dietetic Association Guidelines in pregnancy and International Guidelines for vegetarian and vegan diets; moreover, we also included scientific papers about the nutritional status of mothers, reflecting fetal nutrition and possible detrimental effects on fetal development. We included few data before 2000, only those with crucial results.

Especially, our main goal was to highlight if vegetarian or vegan diets could be considered safe for the mother’s health and for offspring during pregnancy and lactation. We also focused on the effect of these dietary patterns on the lack of micronutrients in order to find a target therapy that could avoid fetal complication. 

## 2. Review

### 2.1. International Guidelines for a Healthy Nutrition in Pregnancy: Omnivores and Vegetarians (see Table S1)

Pregnancy is a critical period during which the mother requires different amount of nutrients for healthy gestation, in order to promote optimal fetal development and to avoid the “reprogramming” of fetal tissue, predisposing the infant to lifelong chronic conditions [26]. Preconception body mass index (BMI) is determinant to avoid adverse outcomes during pregnancy. Overweight and caloric excess are related to maternal and fetal health risk including diabetes, preeclampsia and cardiovascular disease, so gestational weight gain during pregnancy must be kept into the normal range recommended [27]. On the contrary, low BMI and malnourished mothers could impair fetal development and nutrients supply, leading to adverse birth outcomes, physical and cognitive delays in childhood, and metabolic disorders in adulthood [28]. 

In the course of normal gestation plasma volume expands, producing a decrease of vitamins and minerals concentrations; however, plasma lipids and cholesterol arise. During early gestation the fat stores of mothers increase, whereas the last gestation is characterized by increased insulin resistance: these are metabolic changes essential to support fetal growth [29].

According to the 2015–2020 Dietary Guidelines for Americans (DGA), pregnant women have to consume a variety of foods to maintain energy and nutrient requirements and to gain recommended amounts of weight. According to the Dietary Reference Intakes (DRIs) and Institute of Medicine (IOM), caloric necessities are no higher than the estimated energy requirement for nonpregnant women until the second trimester. The extra energy requirement per day is 340 kcal in the second trimester and 452 kcal in the third trimester (IOM), or 260 kcal and 500 kcal, respectively (according to European Food Safety Authority—EFSA) [30,31]. The Guidelines recommend practicing moderate intensity physical activity during pregnancy for benefits to both the mother and fetus [32]. 

All pregnant women need appropriate nutritional supplementation. Iron supplementation is essential for iron deficiency anemia during gestation [33]. Folic acid consumption of 600 mcg/day from fortified foods and dietary supplements is needed to avoid neural tube defects (NTD) [34]. In addition adequate levels of vitamin D (600 UI/day), choline (450 mg/day), and iodine (220 mcg/day) are needed for normal fetal growth and brain development [35,36,37]. During a normal pregnancy the efficiency of calcium absorption increases, so the intake of calcium is equal to a nonpregnant woman of the same age. During pregnancy and lactation, adequate calcium intake is considered to be 1000 mg/day. Women with calcium intakes less than 500 mg/day need additional amounts to achieve maternal and fetal bone requirements [38]. Supplementary Materials Table S1 shows the daily nutrients requirements of the principal macro- and micronutrients during pregnancy and lactation, as well as a comparison between the American Guidelines, IOM, EFSA, and International Federation of Gynecology and Obstetrics (FIGO) [39,40].

The Physicians Committee for Responsible Medicine suggests that vegetarian pregnant women have to follow the recommendations for protein intake and should increase up to 25 g of protein each day to reach 1.1 g/kg/day as in pregnancy the demand of protein increases [40,41]. The recommendations are to consume daily portions of dark green vegetables (1–2 servings), other vegetables and fruits (4–5 servings), bean and soy products (3–4 servings), whole grains (six or more servings), and nuts, seeds, and wheat germ (1–2 servings). Vegetarians should include sources of protein, iron, and vitamin B-12 intake, as well as calcium and vitamin D if avoiding milk products. Charts listed in these guidelines are useful for substituting beans, tofu, nuts, eggs, and seeds for meats, and include alternative food sources for calcium if milk and dairy products are rejected [3,41].

### 2.2. International Guidelines for Vegetarian and Vegan Diets 

According to the Academy of Nutrition and Dietetics a well planned plant-based eating pattern could be appropriate for all stages of life if adequate and healthy recommendations are followed. For example, in the vegetarian population, if the diet includes a variety of plant products, it would provide the same protein quality as a diet that included meat. Venti et al. provided a modified food guide pyramid for vegetarian and vegan diets [42]. According to these recommendations, the normal intake of protein is 0.8 g/kg/day for an adult woman who follows diets containing high quality protein such as egg, meat, milk, or fish. If dairy products, whole grains, beans, nuts, and seeds are the primary protein sources in the diet, the protein digestibility falls, so the recommendation for dietary protein should increase 20% for vegetarian adult woman until 1 g/kg/day. The protein quality of foods is assessed by their Protein Digestibility Corrected Amino Acid Score (PDCAAS), which measures the score for amino acid digestibility. Values close to 1 correspond to animal products supplying all nine essential amino acids, while values below 0.7 are typical of plant foodstuffs. Even if the score is lower, the combination of more vegetable foods with different amino acid composition could enhance the overall quality of their protein component [31].

According to Messina’s guide for North American vegetarians, this population have to consume more legumes, nuts, tofu, beans, seeds, fortified breakfast cereals, milk, cheese or yogurt, and fortified soymilk, which are good sources of vitamin B12, vitamin D, iron, and calcium (the recommendation is the intake of 1200 to 1500 mg/day of calcium, 20% more than omnivores). People who adhere to vegan diets would have to intake daily supplements of vitamin D, vitamin B12, and calcium because the average of these nutrients is insufficient [43].

Although the vegetarian and vegan population tries to compensate the adequate intake of micronutrients, these types of diets limit their amounts. Alfa-linolenic acid (omega-3 or ALA) derives from foods such as flax seeds, chia seeds, mungo beans, walnuts, and canola and soybean oils, so vegetarians consume an abundance of ALA. One teaspoonful of walnuts, soybeans, and mungo beans or one teaspoonful of flaxseed oil or ground flaxseed will supply the daily requirement of ALA. High temperature damages this oil, so it should not be fried. Linoleic acid (n-6 or LA) is derived from nuts, seeds, leafy vegetables, grains, and vegetable oils (corn, safflower, sesame, and sunflower). Unsaturated fatty acids are crucial for cell membrane function and the production of eicosanoids (thromboxanes, leukotrienes, prostaglandins, and prostacyclins). LA (*n*-6) is converted to arachidonic acid (AA) and ALA (*n*-3) is converted to eicosapentaenoic acid (EPA) and docosahexaenoic acid (DHA). High intake of LA (*n*-6) inhibits the synthesis of DHA from ALA (*n*-3). Consequently, a balanced ratio intake of *n*-6 and *n*-3 as 1:2 or 1:3 would be most advantageous for vegetarians. To improve the conversion of ALA to more physiologically active fatty acids like EPA and DHA is essential for the development of brain and nervous system. Vegetarians should guarantee that their diet contains sufficient proteins, pyridoxine, biotin, calcium, copper, magnesium, and zinc. In addition, they should reduce intake of n-6 fatty acids and trans fatty acids that inhibit such conversion, by limiting consumption of processed and deep-fried foods, and alcohol [44]. However, people who adhere to vegetarian or vegan diets steadily show low plasma EPA or DHA, especially vegans compared to nonvegetarians [45]. 

Moreover vegetarian diets have been associated with iron deficiency but not to iron deficiency anemia so the recommendation for vegetarians is to enrich the diet with iron-fortified breads and cereals, beans and lentils, raisins, and blackstrap molasses, as well as sources of vitamin C, like tomatoes and citrus fruits for optimal iron absorption, cooked in cast iron pans [46]. The iron from vegetarian diets is less available for absorption because these diets contain nonheme iron from plants that is worst absorbed than heme iron contained in animal food like meat. In contrast the absorption of nonheme iron in vegetarian people is high to compensate low body iron stores compared to nonvegetarians. The mean iron absorption from a vegetarian diet is estimated 10% compared to 18% of a diet containing meat. Moreover, nonheme iron absorption is inhibited by whole grains, legumes, and nuts because they contain phytic acid so a balanced diet is needed [47]. Zinc deficiency is also common in people who adhere to vegetarian diets due to the inhibition of zinc absorption from plant food with phytic acid, an inhibitor of zinc bioavailability, so the recommendation is a 50% greater intake of zinc [48]. Calcium intake is high in LOV but vegans show low intake of calcium than recommended. Calcium can be found in low-oxalate (high bioavailability) foods such as bokchoi, broccoli, Chinese cabbage, collards, kale, okra, turnip greens, and soy products. Other foods with slightly less calcium bioavailability are fortified soymilk, sesame seeds, almonds, and red and white beans. Calcium bioavailability from plant foods can be affected by oxalates and phytates, which are inhibitors of calcium absorption; for example spinach and rhubarb have a low calcium bioavailability, whereas kale, broccoli, and bokchoi have high calcium bioavailability. Another important calcium source is water, in particular, hard water that has high calcium and magnesium levels derived from groundwater, particularly from limestone and dolomite [31]. If dietary intake of calcium is low, calcium supplemention should be recommended in divided doses [49,50,51].

Low vitamin D is the most common deficiency among LOV and vegan people so they may need sun exposure, food fortified with vitamin D, and daily supplements to maintain adequate serum levels. Good sources of vitamin D are found in fish liver oils, fatty fish, and egg yolks, but vitamin content in these foods varies. However, reaching adequate levels of vitamin D from fortified foods is a challenge for vegans because few plant foods are fortified with this vitamin. In such cases, especially in vegan population, vitamin D supplements seem to be the most adequate way to ensure correct vitamin D status [52]. 

Vegetarian diets are at serious risk of vitamin B12 depletion and/or deficiency, an essential micronutrient that plays a specific role in the synthesis of DNA and red blood cell division and in one carbon metabolism. Vitamin B12 is an essential nutrient that transfers the methyl group in a methionine synthase-requiring reaction, converting homocysteine to methionine. It is essential for the synthesis of energy in mitochondria and for erythropoiesis in the bone marrow. In addition, it is also necessary for the synthesis of myelin and the maintenance of neural axons. Vitamin B12, also called cobalamin, is found in adequate ranges only in animal and dairy foods. If the consumption of animal foods is absent as seen in vegetarian diets it results in low intake and cobalamin deficiency due to its scarce presence in plant foods, although vegetarians consume some fortified foods as cereals and soy products. This deficiency generates hematological alterations, impairment of erythropoiesis, and neurologically-poor outcomes as it plays a key role in neuronal myelinization [53]. The DRI for Vitamin B12 is 2.4 µg/day for adults [54]. The European Food Safety Authority (EFSA) Panel on Dietetic Products, Nutrition and Allergies established an Adequate Intake (AI) of cobalamin nearly 4 µg/day for adults [55]. A high intake of plant-based foods may result in high folate levels, which may cause vitamin B-12 depletion by rectifying the hematologic alterations that are present with vitamin B12 insufficiency. A high prevalence of deficiency, assessed by methylmalonic acid and/or holotranscobalamin II levels, is frequent among vegetarians, and a high prevalence of increased concentration of homocysteine. Vegan subjects reported the most compromised status of vitamin B12 because they do not consume eggs, yoghurt, cheese, and milk, which are natural sources of cobalamin. Chronic low intake of vitamin B12 can lead to a depletion status, and this progressive deficiency may become clinically evident after years, resulting in permanent neurologic damage [56,57,58].

### 2.3. The Effects of Vegetarian and Vegan Diets during Pregnancy on Maternal Nutritional Profile (see Table S2)

Vegetarian and vegan diets have been considered a nutritional challenge during pregnancy, and they require strong awareness to achieve complete intake of essential nutrients, as such, these diets are at risk of nutritional deficiencies. As described, several studies have demonstrated the insufficient supply of essential nutrients such as vitamin B12, vitamin D, calcium, zinc, iron, proteins, essential fatty acids, and iodine in vegetarian and vegan diets [46]. Here we discuss the published evidence of the effect of plant food diets on maternal nutritional profile. The choice of vegetarian or vegan diet is always in preconception, period so well adjusted preconception nutrition is essential for healthy pregnancy. The nutritional pattern depends on the socioeconomic status of the mother, ethnicity, and the reason for choosing vegetarian diets. If the choice is not cultural but is due to ethical beliefs and good socioeconomic status, the probability of a balanced diet increases [59]. In a case–control poblational study called the KOALA Birth Cohort Study (a prospective cohort of 2834 mother–infant pairs in the Netherlands), vegetarian pregnant women who chose organic foods, had higher education and calculated good intake of macro- and micronutrients for a balanced diet [60]. In the same cohort, vegetarian pregnant women had lower BMI if compared to women following a conventional dietary pattern, and lower prevalence of overweight and obesity 4–5 years after delivery [61]. Generally it is difficult to verify the effect of such diets and to separate them from other determinant factors such as ethnicity, lifestyles, or smoking.

#### 2.3.1. Vitamin B12

A recent review shows that there is a high prevalence of vitamin B12 depletion or deficiency among vegetarians [62]. During pregnancy the intestinal absorption of vitamin B12 increases; absorption is better in small amounts and frequent intervals as fetal needs are not high. Vitamin B12 derived from maternal tissue stores does not cross the placenta, but the absorbed vitamin B12 is transferred across the placenta. Low maternal serum concentration of vitamin B12 during the first trimester is a risk factor for NTD and poor maternal outcomes such as preeclampsia, macrocytic anemia, and neurological impairment [63]. The estimated average requirement (EAR) of vitamin B12 is 2.2 µg/day for pregnancy and 2.4 µg/day for lactation [39]. Many people with vitamin B12 deficiency present signs of clinical anemia or mild anemia. The macrocytosis may be masked by a concomitant disorder, such as iron deficiency, thalassemias, or typical high folate levels in vegetarian women [63]. Dietary deficiency of vitamin B12 is frequent in some developing countries or population with marginal status like India, the Middle East, Africa, China, and Central America [64].

In the prospective PREFORM study in Toronto (*n* = 368), the prevalence of suboptimal B12 status (serum total B12 <210 pmol/L) was 35% at 12–16 gestational weeks and 43% at delivery; the prevalence of B12 deficiency (serum total B12 <148 pmol/L) was 17% and 38%, respectively. Maternal dietary vitamin B12 intake during pregnancy was weakly associated with maternal vitamin B12 levels [65]. Another prospective longitudinal study conducted during pregnancy showed that the prevalence of B12 deficiency increased between the second and third trimester from 8% to 35% in healthy pregnant women with B12 intake >RDA (2.6 µg/day). This decrement of plasma total B12 during pregnancy could be the consequence of increased metabolic rate, active B12 transport across the placenta, and hemodilution [66], so it is important to distinguish if very low serum vitamin B12 in pregnancy represents a true deficiency or an exaggerated physiological fall.

Koebnick et al., in a longitudinal cohort study, compared serum vitamin B12 and homocysteine concentrations in pregnant women consuming a LOV diet, low meat diet (LMD = <300 g/wk), or a diet with larger amounts of meat (>300 g/wk). Dietary vitamin B12 intake, serum levels of vitamin B12, and plasma total homocysteine concentrations were measured once in each trimester. This data included 27 pregnant LOV, 43 pregnant low meat consumers and 39 pregnant controls who consumed more meat as a ‘Western diet’ (WD). The following criteria were used to consider “low serum concentration of vitamin B12”; <130 pmol/L in the first trimester, <120 pmol/L in the second trimester, and <100 pmol/L in the third trimester. The prevalence of B12 deficiency, based on these cutoffs in at least one trimester, was found to be 39% of LOV, 9% of low-meat eaters, and 3% of the control group. Also, the odds ratio of having a low serum B12 during at least one trimester was 3.9 (95% confidence interval, 1.9–6.1) times higher among LOV and 1.8 (1.0–3.9) times higher among low meat consumers compared with the odds among women of control group. The deficiency rate for women was 33% in the first trimester, 17% in the second, and 39% in the third trimesters. The limitations of this study are that the sample did not include vegan participants, the inclusion of a small population, and that some comparisons were more cross-sectional than longitudinal due to the study design. Moreover high folate intake and folate supplementation during pregnancy may cover the real effects of low vitamin B12 intake on plasma. The higher deficiency in vegetarian pregnant women in the third trimester pointed out a depletion of vitamin B12 stores instead of expanded blood volume. The authors recommend a higher intake of vitamin B-12 of more than 3.0 mcg/daily for pregnant women consuming a LOV diet [67].

Gibson et al. conducted a cross-sectional study in 99 pregnant women from Ethiopia and included participants whose diet was based on either maize (*Zea mays* L.) and fermented enset (*Enset ventricosum*) products that are the major staple foods consumed. As in the Sidama Zone of Southern Ethiopia, animal products often provide <1% of total energy, this low intake of animal products is coincident with infections and bacterial overgrowth, meaning that pregnant women in Sidama are at high risk of vitamin B-12 deficiency and possibly of folate deficiency. They described that 23% of pregnant women had low plasma vitamin B12 (<150 pmol/L), but 62% had elevated plasma methylmalonic acid (MMA) (>271 nmol/L). None had elevated plasma cystathionine or total homocysteine. Even though they were diagnosed with vitamin B-12 deficiency, there were no signs of macrocytic anemia. This data was limited by the small sample and it was conducted in a specific population [68]. In a cross-sectional study conducted in 284 Canadian pregnant women in Vancouver, the authors found that pregnant South Asian women had suboptimal B12 status (11.5%) or B12 deficiency (61.5%) and showed more MMA plasma concentrations. Plasma total B12 concentration was positively associated with age, education, and B12 supplements. Pre-pregnancy BMI was the strongest independent predictor of plasma total B12 concentration. One limitation of this data was the absence of a dietary assessment to quantify B12 intake, as this specific population promoted vegetarianism [69]. A study conducted in small sample of pregnant Indian women showed that hyperhomocystinemia leads to global DNA hypermethylation in vegetarian population due to low dietary intake of vitamin B-12 during pregnancy and that this could predispose to cardiovascular risk and obesity [70]. All these studies provide evidence that vegetarian and vegan pregnant women are at high risk of vitamin B12 depletion.

On the contrary, a longitudinal case–control study recruited 109 participants and showed that high folate intake in LOV pregnant women and low risk of folate deficiency [71].

#### 2.3.2. Vitamin D and Calcium

Plasma levels of vitamin D during pregnancy depend on sunlight exposure and intake of foods high in vitamin D, fortified foods, or supplements. During pregnancy there is not an increment of vitamin D requirements. Vitamin D deficiencies exist among the general population, especially those with dark skin and vegetarians. Data from the third NHANES indicate that ~42% of African American women and 4% of white women show vitamin D insufficiency [72]. Vegan diets have shown lower average intakes of vitamin D than lacto-vegetarians and omnivores [73]. Sachan et al. performed a cross-sectional data describing the levels of serum vitamin D, calcium, and parathormone (PTH) in 207 urban and rural pregnant women (84.3% of urban and 83.6% of rural women). They found vitamin D values below the cutoff (<22.5 ng/mL) and low calcium intake in the population who does not consume meat. In this study, 14% of the mothers had biochemical osteomalacia [74]. Another data found similar results in Iranian pregnant women at the delivery [75]. Dasgupta et al. recruited a total of 50 pregnant female aged 20–40 years of the Northern Eastern Part of India and assessed vitamin D during the first trimester. Nearly 42% of the cases had vitamin D deficiency and 14% had vitamin D insufficiency in the first trimester; 63.63% who had 25(OH)D levels <20 ng/mL were vegetarians. They did not find any association between vitamin D levels and multivitamin supplements or dietary calcium intake [76]. These studies were limited by the recruitment of a specific population.

During pregnancy and lactation, adequate calcium intake is considered to be 1000 mg/day. Vegetarians and vegans should consume 1200 to 1500 mg/day of calcium, 20% more than omnivores. Pregnant and lactating women should consume a minimum of eight servings of calcium-rich foods [12].

Despite recommendations about a good intake during pregnancy, vegetarian pregnant women are at high risk of vitamin D deficiency and may suffer bone impairment, osteoporosis, and hypocalcemia [77]. In a population-based cross-sectional survey conducted in Shaanxi Province in Northwest China, pregnant women who followed a vegetarian pattern had low calcium intake [78].

#### 2.3.3. Iron

During pregnancy, mild anemia is physiologic as a consequence of the normal hemodilution status. During the second and third trimesters of pregnancy there is a rise in maternal blood volume and iron transportation to the placenta and fetus, showing an increased necessity for iron. Iron absorption from plant (nonheme) and animal (heme) supply is improved during pregnancy and increases with each trimester. Inhibitors of iron absorption include calcium, coffee, and fiber. Vitamin C can help to enhance absorption by reducing the inhibitory effects of phytate. [79,80]. The average of dietary iron needed in pregnancy is dependent on maternal preconception stores and estimated requirements are shown in Table S1.

A systematic review and meta-analysis showed that vegetarian population exhibits lower stores of iron compared to nonvegetarian [81]. In pregnant vegetarian women the results are controversial. A British study, conducted in a cohort of 1274 pregnant women aged 18–45 years, showed that vegetarians had adequate iron intakes from diet and they followed the recommended iron supplementation during the first and second trimesters of pregnancy more than nonvegetarians [82]. Sharma et al. described a high prevalence of anemia in Indian pregnant women because of very low frequency of meat eating. However, this study was cross-sectional and limited to a specific population [83].

#### 2.3.4. Essential Fatty Acids

LA (*n*-6) and ALA (*n*-3) are polyunsaturated fatty acids which are obtained from food sources. LA (*n*-6) is converted to arachidonic acid (AA), and ALA is converted to EPA and DHA. DHA is an important component of neural and retinal membranes. It accumulates in the brain and retina during the end of gestation and early postnatal life. Polyunsaturated fatty acids are transferred via the placenta to the developing fetus from the mother’s plasma. Adequate provision of DHA is thought to be essential for optimal visual and neurologic development during early life [84]. A significant percentage of women of childbearing age in Europe are vegetarian and the majority of the population in countries such as India. This group is particularly at risk as the exclusion of meat or fish from the diet may determine very low intakes of DHA. According to a more comprehensive database and improved questionnaire, vegetarian women can achieve higher intakes of DHA (30 mg/day) [85]. For example, women consuming diets with marine products or eating marine oil supplements can reach very high intakes of DHA (>1000 mg/day). The adequate ingestion of DHA is especially important in the last stage of pregnancy as placenta is able to channel the uptake of DHA to the fetus [86].

The literature regarding DHA levels in pregnancy is limited. Pregnant and lactating women increase the necessity of a source of preformed DHA. Lower proportions of DHA have been found in the fetal plasma of vegetarian mothers if compared with mothers who are omnivores [87]. 

#### 2.3.5. Zinc

Zinc is less bioavailable and it is likely to be present in lower ranges when obtained from plant-derived compared to animal food sources [47]. Vegetarians usually have lower zinc consumption compared with omnivores and their serum zinc levels are lower but in the normal range [88].

During pregnancy the need of Zinc increases so women are encouraged to enhance the intake of zinc and adopting food preparation methods which improve its absorption (soaking, germination fermentation, and sourdough leavening of bread) and reduce phytate levels in zinc rich foods [89].

Although high zinc intake is essential during pregnancy, the consequences of zinc deficiency are not described, so it has been postulated that the body adapts the absorption according to the average intake [90]. The current recommendation of dietary intake for zinc in pregnant women aged 19–50 years is 11 mg/day [48]. A meta-analysis compared the dietary zinc intake of pregnant vegetarian and nonvegetarian (NV) groups: the zinc intake of vegetarians was found to be lower than that of NV (−1.38 ± 0.35 mg/day; p < 0.001); the exclusion of low meat eaters from the analysis revealed a greater difference (−1.53 ± 0.44 mg/day; *p* = 0.001). Neither vegetarian nor NV groups met the recommended dietary allowance (RDA) for zinc. In contrast, the evidence evaluated in this systematic review suggests that there is no difference between groups in biomarkers of zinc status (concentrations of zinc in serum/plasma, urine, and hair) or in functional outcomes associated with pregnancy (period of gestation and birth weight) [91].

#### 2.3.6. Iodine, Magnesium

Vegetarian or vegan diets may result in low iodine intake because the main dietary sources of iodine are meat, fish, and dairy products, but iodine in the salt could avoid the risk of deficiency [92]. 

An adequate magnesium status during pregnancy is essential for fetal development. Serum magnesium levels physiologically decrease during pregnancy due to high demand, higher renal excretion, and haemodilution. In a longitudinal study conducted in 108 healthy pregnant women, significant higher dietary magnesium intakes were observed in pregnant women consuming a plant-based diet (508,714 mg/day for LOV and 504,711 mg/day for low-meat eaters) than in pregnant women consuming a control diet (41,279 mg/day). Urinary magnesium excretion was higher in LOV, followed by low-meat eaters, when compared to the control group [93]. So vegetarian or vegan diets result in high magnesium levels.

#### 2.3.7. Proteins

Proteins demand during pregnancy and lactation increases up to 71 g/day (1.1–1.2 g/kg/day) compared to 46 g/day (0.8 g/kg/day) for nonpregnant women. Protein deposition in maternal and fetal tissues increases throughout pregnancy, much more during the third trimester. Enhanced protein synthesis occurs, combined with decreased amino acid catabolism and urea synthesis. The increment of circulating plasma amino acids is a conservation mechanism for retention of protein during a period of increased demand. Additional protein is needed in pregnancy to reach the estimated 21 g/day deposited in fetal, placental, and maternal tissues during the second and third trimesters. Proteins derived from plants are sufficient to reach these needs. Legumes, nuts, tofu, and eggs are good sources of proteins. Soy protein can reach adequate protein needs as animal protein. Cereals content is low in lysine, so this essential amino acid can be acquired by the intake of more beans and soy products. An increase in all sources of protein can compensate a low lysine average [63]. Pregnant women who adhere to vegan diet are at higher risk of protein deficiency so additional protein is indicated for the vegan diet in the second and third trimesters: 25 g of protein may be added by including 1.5 cups of lentils or 2.5 cups of soy milk per day [94].

### 2.4. The Effects of Vegetarian and Vegan Diets during Pregnancy on Mother’s Health (see Table S3)

#### 2.4.1. Preeclampsia (PE)

Studies about the effects of vegetarian and vegan diet on maternal outcome have yielded incongruent results. The most ancient retrospective data conducted by Carter et al. [95], in 775 vegan mothers in “the Farm”—a vegan community in Summertown, Tennessee—reported only one case of preeclampsia (0.13%).

PE may be caused by a relative prostacyclin deficiency secondary to an excessive production of thromboxane A2. A vegan diet (low in AA) might provide protection against this condition, especially if the conversion of LA to AA is inhibited by decreased activity of the enzyme delta-6-desaturase. Another observational prospective study enrolled 1.538 pregnant Washington state residents and used food frequency questionnaire (FFQ) to assess maternal dietary intake three months before and during pregnancy [96]. They reported that dietary fiber might reduce dyslipidaemia, a condition related to PE. In the group of fiber intake the relative risk (RR) of PE for women in the highest (≥21.2 g/day) vs. the lowest quartile (<11.9 g/day) was 0.28 (95% CI = 0.11–0.75). The same was for water-soluble fiber and insoluble fiber.

Vegetarian diet combined with physical activity seems to reduce the risk of PE. A longitudinal study conducted in 238 black pregnant women from Congo showed that the incidence of arterial hypertension was 4.6% overall (2.9% of whom had PE and 1.7% of whom showed transient hypertension). Women who did not usually consume daily servings of vegetables during pregnancy (33.3%) showed higher frequency of PE compared to women who consumed more than three servings of vegetables per day (3.7%) [97]. In a case–control study, conducted in 172 preeclamptic pregnant women and 339 normotensive controls, the authors described that foods that were beneficial in decreasing the risk of preeclampsia were fruits, vegetables, cereals, dark bread, and low-fat dairy products for their high content of fiber, calcium, and potassium [98]. Despite the evidence support the protective effect of plant based diets on PE, these results might be read with caution because PE is a multifactorial condition.

#### 2.4.2. Gestational Diabetes

Vegetarian diets and high intake of fiber could avoid the development of Gestational Diabetes (GD). In a prospective cohort study of 13,110 pregravid women of Nurses’ Health Study II (USA) the authors conducted a self-reported FFQ. They described 758 cases of GD in an 8-year period. Dietary total fiber and cereal and fruit fiber were negatively associated with GD risk after adjustment. Each 10 g/day increment in total fiber intake was associated with 26% (95% CI 9–49) reduction in GD risk. Each 5 g/day increment in cereals was linked to a 23% (9–36) risk reduction and fruit fiber with a 26% (5–42) risk reduction. The combination of high-glycemic load and low-cereal fiber diet was linked to a 2.15-fold (1.04–4.29) increased risk of GD [99]. Another randomized controlled clinical trial included 52 women with GD who were randomly assigned to receive control (*n* = 26) vs. DASH (Dietary Approaches to Stop Hypertension) diet (*n* = 26) only for four weeks. The control diet was composed of 45–55% carbohydrates, 15–20% protein, and 25–30% fat. The DASH diet was rich in fruits, vegetables, whole grains, low-fat dairy products, lower saturated fats, cholesterol, and refined grains with a total of 2400 mg/day of sodium. In addition, the DASH diet is low energy-dense and contains high amounts of dietary fiber, phytoestrogens, potassium, calcium, magnesium, and folic acid. Women who consumed the control diet had more incidence of cesarean section—46.2% vs. 80.8% of control group—as well as less need for insulin after intervention: 23% vs. 73% for control group [100]. Assaf et al. [101] conducted a case–control study of 697 normoglycemic women who were randomized (at 8–12 weeks of gestation) in control group (337), of which fat consumption was limited to 30% of total caloric intake, or the intervention group (360), where a MedDiet was offered, enhanced with Extra Virgin Olive Oil (EVOO) and pistachios (40–42% fats of total caloric intake). The composite outcome was defined as having one event of emergency C-section, perineal trauma, pregnancy-induced hypertension and preeclampsia, prematurity, large for gestational age (LGA), and small for gestational age (SGA). The authors found a significant reduction in the risk of composite outcome in the group who received the MedDiet enhanced with EVOO and nuts. The data involved an Indian population and found that Indian vegetarians display a high prevalence of GD; however this concern is of a specific population with high susceptibility to diabetes [102]. Moreover a cross-sectional study in India enrolled 995 white British pregnant women, described that low circulating levels of vitamin B12 were correlated to high maternal BMI and high insulin resistance (assessed by HOMA method) [103]. 

Nevertheless, a recent Cochrane review showed that dietary interventions in pregnancy for preventing gestational diabetes mellitus need more high-quality evidence to determine their potential effects in pregnancy [104]. In addition, according to a systematic review of observational studies on gestational weight gain (GWG), the intake of carbohydrates and vegetarian diets was associated with lower GWG, contrary to diets with higher intakes of proteins, animal fats, and energy-dense foods, suggesting that plant-based dietary patterns could be beneficial in preventing GWG and consequently GD [105].

#### 2.4.3. Preterm Delivery

Data correlating preterm delivery and vegetarian diets are controversial. A retrospective study conducted in Australia analyzed maternal dietary patterns in the 12 months before conception on fetal growth and preterm delivery [106]. They studied three patterns: (1) high-protein/fruit (characterized by fish, meat, chicken, fruit, and some whole grains); (2) high-fat/sugar/takeaway (takeaway foods, potato chips, and refined grains); and (3) vegetarian-type (vegetables, legumes, and whole grains). A 1-SD (standard deviation) increase in the scores on the high-protein/fruit pattern was associated with decreased likelihood of preterm birth, whereas the reverse direction was apparent for the high-fat/sugar/takeaway pattern. A 1-SD increase in the scores on the high fat/sugar/takeaway pattern was also associated with shorter gestation and low birth length. A longitudinal prospective study conducted on Danish National Birth Cohort enrolled 35,530 nonsmoking women, investigated the association of maternal intake of a Mediterranean-type diet (MD) assessed by FFQ and preterm birth [107]. MD was defined as fish twice a week or more, use of olive or rape seed oil, consumption of five fruits and vegetables a day, ate meat (other than poultry and fish) at most twice a week, and drank at most two cups of coffee a day. For early preterm birth Odds ratio was 0.61 (95% CI: 0.35–1.05) in women fulfilling all criteria for MD and 0.28 (0.11–0.76) in women with none, adjusted for possible confounders (parity, BMI, maternal height, socioeconomic status and cohabitant status). So MD during pregnancy may reduce the risk of early delivery in Danish women. Data including the Norwegian Mother and Child Cohort Study (MoBa) enrolled 26,115 pregnant women using FFQ and divided them in three groups: 569 of full criteria of MD, 25,397 1–4 criteria of MD, and 159 none criteria. No differences were found in preterm birth (OR: 0.73; 95% CI: 0.32, 1.68). A significantly lower rate of preterm birth was found in women with fish intake twice or more a week (OR: 0.84; 95% CI: 0.74, 0.95) [108]. A randomized clinical trial conducted in Norway on 290 pregnant White women (BMI 19–32), analyzed the effect of a cholesterol-lowering diet on maternal, cord, and neonatal plasma lipids and pregnancy outcomes [109]. Maternal total and low-density lipoprotein (LDL) cholesterol levels were lowered in the intervention compared with the control group, but lipid levels in cord blood and in neonates born to mothers in the intervention versus the control groups did not differ. Preterm birth (< 37 weeks) was lower in the intervention group (RR 0.10; 95% CI 0.01–0.77). In addition a prospective data performed in Denmark found the relationship of low consumption of seafood with a strong risk of preterm delivery and low birth weight, OR = 3.6 (95% confidence interval 1.2 to 11.2) showing that low plasma concentration of EPA and DHA during pregnancy is a strong risk factor for subsequent early preterm birth in Danish women [110,111].

#### 2.4.4. Consequences of Unbalanced Nutrition on Mother’s Mental Health

Recently, maternal depression has been linked to inadequate nutrition during pregnancy. Pregnant women are particularly vulnerable to the adverse effects of poor nutrition on mood because pregnancy and lactation increase nutrient requirements. Plausible links between nutrition and mood have been reported for folate, vitamin B12, calcium, vitamin D, iron, selenium, zinc, and PUFAs, which are required for the biosynthesis of several neurotransmitters such as serotonin, dopamine, and norepinephrine [112]. Numerous studies have found a positive association between low *n*-3 levels and a higher incidence of maternal depression. The two *n*-3 fatty acids most correlated to brain development and function are EPA and DHA, of which, the latter is the most prominent in the brain. Nevertheless the current evidence from randomised controlled trials is inconclusive [113]. A recent study assessed that self-reported postpartum depression was more prevalent among vegetarians than omnivorous subjects, probably due to inadequate micronutrients intake [114].

### 2.5. The Effects of Vegetarian and Vegan Diets during Pregnancy on Fetal Outcomes (see Table S4)

Fetal outcomes depend on a balanced interchange between maternal nutrients, placental transport and fetal growth factors. Maternal undernutrition may lead impairment of fetal development due to nutrient limitations and decreased nutrient source for fetal growth, changes in placental functions, and epigenetic modifications in the fetal genome. Macro- and micronutrients may directly regulate DNA stability and phenotypic adaptation by influencing the availability of methyl donors thereby modulating epigenetics mechanisms. In animal models, the metabolism of amino acids (glycine, histidine, methionine, and serine) and vitamins (B6, B12, and folate) provide methyl donors for DNA and protein methylation, emphasizing the critical role of balanced nutrient supply through placenta [115]. 

Dietary patterns may involve very different foods that contain a multitude of nutrients with interactive functions. Many of them are also correlated, so it is difficult to separate their effects. Thus, the cumulative effects of nutrients may be easier to identify than those of a single nutrient. 

The increase of women adopting vegetarian or vegan diets has raised concerns about these risks during pregnancy. Nevertheless, the effect of vegetarian or vegan diets on fetal anthropometrics differs considerably between studies. Many studies have found positive correlation between birth weight (BW) and maternal intake of certain food items such as milk, fruits, and green leafy vegetables [116,117]. A recent review included papers that assessed diet by FFQ and studied the association among different dietary patterns and BW. Authors found that the patterns positively associated with BW were labeled “nutrient dense”, “protein-rich”, “health conscious”, and “Mediterranean”. Those negatively associated with BW were labeled “Western”, “processed”, “vegetarian”, “transitional”, and “wheat products”. The dietary patterns “Western” and “wheat products” were also associated with higher risk of SGA babies, whereas a “traditional” pattern in New Zealand was inversely associated with having a SGA baby [118]. Wen et al. [119] found an association between vegetarian diet and lower fetal growth during the second trimester. In a prospective data reported by Reddy et al. [120], birth weight and head circumference and length were lower in the infants born from South Asian vegetarians, even after adjusting for maternal height, duration of gestation, parity, gender of infants, and smoking habits. A systematic review of the literature performed by Murphy et al. [121], studied the associations of consumption of fruits and vegetables during pregnancy with infant birth weight or SGA births. This data identified limited evidence of a positive association between fruit and vegetable consumption during pregnancy and birth weight. 

On the contrary, there are data demonstrating a protective effect of plant-based dietey patterns on anthropometric fetal development explained by the high content of vitamins in such diets. A case–control study enrolled 787 pregant women and assessed fruit and vegetable consumption during pregnancy using FFQ (in-person interview). The authors reported that women with lower intake of vegetables during the first trimester had higher incidence of SGA and no association was found between fruit consumption and birth outcomes [122]. Four multiethnic birth cohorts in Canada (the NutriGen Alliance) involving 3997 pairs of full-term neonates and mothers assessed dietary patterns by FFQ. Among White Europeans, a plant-based dietary pattern was inversely associated with birth weight and increased risk of SGA. Not adjusted for cooking methods (among South Asians) maternal consumption of a plant-based diet was associated with a higher birth weight [123]. The heterogeneus results may be due to the different dietary context between developed or developing Countries and most of the included studies concerning low BW provided no maternal information on body mass index or gestational weight gain for vegetarians or controls. Other prospective studies showed that average birthweights of infants born from vegetarian mothers do not differ significantly from omnivorous mothers. Clinical relevance of these data is at least doubtful and the heterogeneity of the results suggests the presence of confounding factors. As fetal growth is directly affected by maternal protein intake, pregnant women need to consume an optimal variety of food plants in vegetarian or vegan diets to achieve the same biodisponibility of proteins of omnivorous population as several studies have demonstrated [124,125,126]. 

Micronutrients such as vitamin B12 and zinc are deficient in vegetarian and vegan diets, as the main source are animal products [127]. It has been suggested that the supposed lower BW observed among infants born from mothers who adhere to vegetarian diets may be related to poor nutritional status with regard to iron or vitamin B12. As is highlighted in Fikawati et al.’s longitudinal study, infants may be born as well with low vitamin B12 stores if the maternal intake during pregnancy is inadequate [124]. Maternal B12 status is a key determinant of infant B12 status and is an independent factor for NTD [128]. A recent review demonstrated an association between vitamin B12 status, low BW and preterm delivery [129]. The effects of vegetarian diet on the development of NTD are contradictory [130,131]. Although the direct effect on fetal development has not been well described, vegetarian women may include extra sources of vitamin B12 in their diet to avoid fetal depletion.

Iron deficiency during pregnancy has been associated with low BW and neonatal anemia [132]. Iron derived from meat has better bioavailability than iron provided by plants, therefore vegetarian and vegan women should take a higher content of iron to avoid depletion of internal stores [94]. In fact, a British study found that iron deficiency during pregnancy was not higher in vegetarians and that iron intake was higher in vegetarian women during the first trimester [82], probably due to an awareness of risks and a consequent well planned diet. A recent review showed that vitamin D deficiency is also linked to fetal poor growth and vegetarians are at risk of vitamin D deficiency [133].

An increased risk of hypospadias has been associated to vegetarian or vegan diet during pregnancy, based in the increased intake of phytoestrogens. It was first reported in one prospective study, which obtained an adjusted OR of 4.99 (2.10–11.88) in vegetarian women compared to their omnivorous counterparts. However, this work failed to demonstrate a relationship between the incidence of hypospadias and the intake of soya products, which were supposed to be the first source of phytoestrogens in this lifestyle [134]. Another Scandinavian prospective case–control study reported an increased risk of hypospadias in women with diets excluding meat and fish diet. The study was based in the hypothesis that the lack of some essential amino acids may produce a deficiency during organogenesis [135]. However, these large and well-designed studies had the limitation that their conclusions were based on indirect correlations derived from retrospectively self-administered questionnaires. On the contrary, more recent works, which have focused on the relationship of nutrients to estrogen metabolism and the quantity of phytoestrogens in the diet to hypospadias, have not found positive correlation [136,137]. Furthermore, one Italian study found a positive correlation between hypospadias and a frequent consumption of fish during pregnancy [138]. This contradictory data, together with other studies, suggest that the imbalance of some nutrients or the intake of food contaminants may play roles in the genesis of hypospadias; however, at this point in time, there is not enough information to conclude that vegetarian or vegan diets increase the risk of disruption during the genital organogenesis [139,140].

Plant-based and vegetarian diets, characterized by a high consumption of fruits and vegetables and low or no intakes of cured meat and smoked fish, which represent the principal exogenous dietary sources of nitrate, nitrite, and N-nitroso compounds, associated with a higher risk of developing NTD, would be protective to the risk of congenital malformations [92]. However, it has been described that pickled vegetables are also a source of nitrite and NOCs. A study showed the association between maternal periconceptional consumption of pickled vegetables and NTD in four Chinese countries [141]. They found that >6 pickled vegetable meals/wk increased the risk of NTD, compared with less frequent (i.e., <1 meal/week) consumption. In addition, the authors showed that maternal intake (>1 meal/week) of meat, eggs, or milk had protective effect against the risk of NTD, so the literature is controversial.

### 2.6. The Effects of Vegetarian and Vegan Diet on the Composition of Human Milk

Vegetarian diets differ between individuals: without good information and well planned diets, vegetarians may be at risk for some deficiencies during pregnancy and consequently during breastfeeding period. In the Table S1 we recollected the daily nutrient requirements during pregnancy and lactation. Although vegetarian diets are usually rich in carbohydrates, the need to develop energy reserves during lactation increases demand for more calories. Both vegetarian and nonvegetarian mothers need caloric reserves to reach sufficient energy average for breastfeeding during the postpartum period [142]. The composition of human milk changes dynamically and can vary according to many maternal factors, such as diet and nutritional status. A recent study analyzed the association of maternal nutrition and body composition with human milk composition. They did not find a significant statistical relationship between human milk composition and nutrients in women’s diet during the first six months of lactation. For women in the third month postpartum, they observed moderate to strong correlations between total protein content in milk and the body composition measures such as percentage of fat mass, fat-free mass, and muscle mass. The variance in milk fat content was related to body mass index (BMI), with a significant positive correlation in the first month postpartum. These findings suggest that maternal body composition may be associated with the nutritional value of human milk, with independence of type of diet [143]. Nutritional supplements do not change milk composition in observational studies maybe due to compensatory physiological mechanisms that conserve stable milk macronutrient composition related to the nutritional modifications of maternal diet [144]. Also protein concentration in human milk does not vary in relation to maternal intake of vegetal or animal proteins [145]. Nevertheless, Agostoni et al. [146] reported that animal proteins, compared to plant proteins, are associated with positive psychomotor development indices for infants and young children.

Vegetarian mothers might have low pre-pregnancy nutritional status that can lead to low maternal fat stores for lactation. Fikawati et al. [147] conducted a longitudinal data on mother–infant breastfeeding pairs in Indonesia. They followed 42 pairs of vegetarian and 43 pairs of nonvegetarian. Sociodemographic characteristics did not differ between the two dietary groups except in maternal parity. Vegetarian mothers had lower pre-pregnancy BMI but higher pregnancy weight gain compared to nonvegetarian. This study showed that vegetarian mothers had significantly lower BMI during lactation than nonvegetarian and the breastfeeding had no effect on infant weight and length but had significant effect on mothers’ BMI and weight loss. The mothers in the nonvegetarian group in this study had a significantly greater energy intake compared with the vegetarians. Without adequate intake of energy during lactation the postpartum nutritional profile of vegetarian mothers decreased, so the maternal nutritional stores are sacrificed to support infant normal growth. 

Low maternal vitamin B-12 intake during lactation can lead to low vitamin B12 content in breast milk which can cause permanent neurological disabilities in infants with low vitamin B12 levels. Maternal vitamin B12 status is the major factor affecting the severity of cobalamin deficiency in breast-fed infants and vegetarian and vegan population are at increasing risk [148]. In regions where animal source food consumption is low and prenatal supplementation is not common, infants are at risk of vitamin B12 deficiency, as shown in prospective data which found that maternal and infant serum/plasma and breast milk total vitamin B12 concentrations were significantly higher among Canadian mothers compared to Cambodian mothers [149]. Breastfed infants of mother who adhere to vegan diets are at increased risk of vitamin B12 deficiency [150]. Pawlak et al. [151], conducted a cross-sectional study in which 74 vegan, vegetarian, and nonvegetarian women were recruited. The prevalences of low vitamin B-12 (<310 pmol/L) were 19.2% for vegans, 18.2% for vegetarians, and 15.4% for nonvegetarians, which was independent of diet. Approximately 85% of participants categorized as having low vitamin B-12 were taking vitamin B12 supplements at doses in excess of the Recommended Dietary Allowance but this study had limitation of small sample.

Specific fatty acids that form the total lipid fraction are influenced by maternal nutrition. Fatty acids intake derives from the maternal plasma, or they synthesized endogenously by the mammary glands. Both of these sources are influenced by maternal diet composition [143].

The breast milk of vegetarians and particularly vegans in the United Kingdom showed lower DHA levels if compared to omnivores, but in the United States, vegetarians do not have lower levels of DHA in breast milk lipids, probably due to higher ingestion of ALA from soybean oil or preformed DHA [63]. Compared to nonvegetarians, the breast milk of vegetarians was found to have more than twice the amount of LA and ALA, but less than half the amount of DHA [84]. The levels of DHA in human milk augment with DHA supplementation. Supplementation with DHA during lactation is more useful to raise DHA content in breast milk than supplementing only during pregnancy [152]. A recent review analyzed 13 low- and middle-income countries and showed that the content of DHA in breast milk was very low in populations living mainly on plant-based diets but higher in fish-eating countries [153].

A cross-sectional observational study of 74 lactating women following a vegan (*n* = 26), vegetarian (*n* = 22), or omnivore (*n* = 26) diet pattern was conducted in the United States [154]. They found that breast milk from vegans had significantly higher unsaturated fat and total omega-3 fats, and lower saturated fats, trans fats, and omega-6 to omega-3 ratios than vegetarian and omnivore. DHA concentrations in breast milk were low regardless of maternal diet pattern, and were reflective of low seafood intake and supplement use. Supplement use was relatated to high DHA levels. The study was limited to small sample. The vegan diet contains essentially no ARA and DHA, as plants thought to have fatty acids (such as hempseed, rapeseed, flaxseeds, soy beans, and walnuts) are poorly converted into DHA by the body [155].

In addition, in the developed world, vitamin D deficiency is most frequently diagnosed exclusively in breastfed infants of vegetarian or vegan mothers [156].

More data are needed to evaluate the milk composition of mother following vegetarian or vegan diets.

### 2.7. Vegetarian and Vegan Diet during Pregnancy and Lactation: Target Therapy and Health Intervention 

Specific dietary interventions before, during, and after gestation that are aimed in improving diet quality and setting appropriate intakes of macro- and micronutrients are important to avoid maternal health impairment with consequent physical and neurological fetal abnormalities.

The Mediterranean diet pattern, characterized by low total fat (<30% of energy), low saturated fat (<10% of energy), high complex carbohydrates (but relatively low total carbohydrate), and high dietary fiber intake, is generally considered healthy for all people, and has been recommended as a good preconception and pregnancy diet. The intake of pulses, green leafy vegetables, cereals, and fruit that is associated with best adherence to the Mediterranean diet provides a relatively high intake of folate, which is particularly important in the preconception period. Following a Mediterranean diet also increases the likelihood of achieving adequate intakes of zinc, B vitamins, vitamin A, vitamin E, magnesium, and vitamin C [40].

A strict plant-based diet is suitable during pregnancy but it must be well planned in order to provide all the energy requirements and meet critical nutrients, such as protein, fiber, omega-3, fatty acids, zinc, iodine, calcium, vitamin D, and vitamin B12. The vegetarian-type pattern is not associated to any outcome as preterm birth, BW or SGA if requirements are met [106,127]. Educational resources and food recommendations help vegetarians to consume adequate and complete diets. Some tools could be used for educating vegetarian patients as recording dietary intake by food diaries, providing charts that list of different sources of nutrients or sample menus [63]. Providers should regularly assess a woman’s diet and her energy intake [121]. Guidelines on what to eat during pregnancy and lactation are essentially the same for vegetarians as for meateaters, but women who choose restricted diets may need to consume supplements or fortified food in order to reach the recommended requirements [9].

#### 2.7.1. Proteins

Protein needs are particularly high during pregnancy and lactation. Although protein requirements are easily met in a vegan diet, it is recommended an increase by 10% of protein intake during pregnancy [157]. Adding 1.5 cups of lentils or 2.5 cups of soy milk per day could cover additional protein need [94]. Essential amino acids can be full obtained from both grains and legumes. An increase in all sources of protein can compensate for low lysine intake [63]. Lysine is an amino acid that can be more readily obtained from beans and soy and less readily obtained from cereals. 

Some plant foods, such as soy, lupines, spinach, pseudocereals (buckwheat, quinoa, and amaranth), and hemp seeds, have all the essential amino acids in similar proportions to animal foods. But some other foods, such as antinutritional factors or fiber, interfere in the absorption of plant proteins. Plant proteins have low digestibility (on average 85%) [157]. If protein consumption in a vegan diet is well planned, no differences in infant birth weight in vegan mothers in comparison with omnivorous mothers have been observed [16]. Balanced energy and protein supplementation reduce the risk of stillbirth and SGA, but high-protein supplementation is not recommended [158]. Plasma quantization of amino acids could be performed in a laboratory to detect deficiencies [159]. 

#### 2.7.2. Fibers

Fiber intake is recommended in pregnancy because it improves the richness of microbiota and avoids constipation but an excessive consume can difficult the meeting of appropriate nutrient and energy absorption. Therefore, during second and third trimester, alternative foods are preferred (fruits and vegetables, refined grains, peeled beans, and high-protein, high-energy, fiber-free foods such as soy milk, tofu, and soy yoghurt) [157].

#### 2.7.3. Fatty acids/Omega-3

Due to the limited vegetable sources of DHA, pregnant vegetarian women are encouraged to consume an algae-based supplement. ALA is the only *n*-3 fatty acid present in plant foods (flaxseeds, hempseeds, chia seeds, walnuts, and their oils), but ALA elongation to EPA and DHA is limited, and influenced by diet. High dietary LA intake, excessive intakes of trans fatty acids, inadequate intakes of energy, protein, pyridoxine, biotin, calcium, copper, magnesium, or zinc can impair EPA and DHA synthesis [16]. A balanced intake of *n*-3 and *n*-6 fatty acids is important in order to produce sufficient amounts of DHA and EPA. Vegans and vegetarians can be at a disadvantage in balancing this ratio, because they may limit sources of ALA (n-3) or DHA in their diet, and they typically consume an abundance of LA [63]. Good plant sources of omega-3 fatty acids include ground flaxseeds and flaxseed oil, ground chia seeds, and walnuts. Seed oils rich in omega-6, trans fats (margarine), and tropical oils (coconut, palm, and palm kernel oils), which are rich in saturated fats, should be avoided in order to maintain an optimal omega-6/omega-3 ratio and favor the conversion of ALA into polyunsaturated fatty acids (PUFAs). Olive oil has a low influence in this ratio and, in addition to flaxseed oil, and should be the only additional oil to use as omga-3 source. In a recent Cochrane review, the authors studied supplement of omega-3 fatty acids in pregnant women. They found increased prolonged gestation > 42 weeks from 1.6% to 2.6% in women who received omega-3 LCPUFA compared with no omega-3 (moderate-quality evidence); reduced risk of perinatal death (moderate-quality evidence); fewer neonatal care admissions (moderate-quality evidence); reduced risk of low BW babies (15.6% versus 14% (high-quality evidence)); and a small increase in LGA babies (moderate-quality evidence). PE might possibly be reduced with omega-3 LCPUFA but with low-quality evidence. No differences were found for SGA or intrauterine growth restriction. It seems reasonable to recommend a daily supplementation of DHA for all pregnant women [160]. Even at the pregestational level, an intake of adequate amounts of n–3 PUFAs and DHA plays a major role; indeed, small variations in the habitual maternal dietary composition before pregnancy are likely to be more effective in improving the delivery of long-chain PUFAs to the fetus than large dietary changes in the late stages of pregnancy [92]. In these stages, a daily intake of 100 to 200 mg microalgae-derived DHA supplement is suggested [157].

#### 2.7.4. Zinc

Vegetarians should be encouraged to consume more dietary zinc than the Population Reference Intake (PRI) suggested for omnivores, especially when the dietary phytate/zinc ratio is high [16]. Legumes, soy, nuts, seeds, and grains are all rich in zinc [157]. Zinc absorption can be improved by adopting food preparation methods (soaking, germination fermentation, sourdough leavening of bread) that reduce phytate levels in zinc rich foods. Zinc-fortified foods (e.g., breakfast cereals) can also be used. Zinc-rich foods should be eaten together with foods that contain organic acids such as fruit, and vegetables of Brassicaceae family [16] in order to improve zinc absorption.

#### 2.7.5. Iodine

Vegan diets could provide low intake of iodine, although iodine deficiencies are quite uncommon in Western countries. Iodized salt is the safest way to reach iodine requirements in vegan pregnant and lactating women. Iodized salt varies among countries. The estimated average requirement for iodine in pregnant women is 200 μg/day [157]. Higher salt intake during pregnancy in vegans is considered harmless because of the low incidence of hypertension in this population, and can facilitate to meet the iodine requirements. Another option can be an algae-derived supplement. 

#### 2.7.6. Iron

Iron supplementation is only recommended if iron status has been shown to be low by appropriate blood tests [16]. A well planned vegan diet can meet iron needs. Iron content in vegan diets is in nonheme form, with lower bioavailability than heme-forms from animal sources (1–34% and 15–35%, respectively). During pregnancy it is recommended a daily consumption of iron rich foods as soy, beans, seeds, nuts, and green leafy vegetables as well as vitamin-C in combination. Some cooking considerations and food preparations should also improve iron absorption [157].

#### 2.7.7. Calcium

A vegan diet can protect calcium stocks by increasing absorption [94]. Therefore, supplementation is rarely needed. Vegetarians and vegans should consume 1200 to 1500 mg/day of calcium, which is ~20% more calcium than that recommended for omnivores [63]. Ideally, low-oxalate foods (high bioavailability), such as broccoli or bokchoi, must be preferred. Other foods with slightly less calcium bioavailability are fortified soymilk, sesame seeds, almonds, and red and white beans. Another important source of calcium is hard water. Most of the varied diets above 2400 kcal should completely cover calcium needs [157]. Villar et al. [161] conducted a randomized clinical trial in 8325 pregnant women with dietary calcium intake <600 mg/day. In the intervention group the intake was 1.5 g/day calcium. They did not find differences in PE. They found a reduction of severe preeclamptic complications index. Preterm delivery was reduced only in women <20 years. Neonatal mortality rate was lower in the calcium group. Another data from Cochrane database evaluated calcium supplementation (≥1 g/day) during pregnancy in 13 studies. This supplement was associated with a significant reduction in the risk of preeclampsia, particularly for women with low calcium diets but the significant reduction in the risk of PE associated with calcium supplementation might be overestimated (small effect, high heterogeneity and publication bias) [162]. The World Health Organization recommends 1.5 g to 2 g of calcium daily for pregnant women with low dietary calcium intake [40].

#### 2.7.8. Vitamin D

Vegans must rely strongly on ultraviolet B rays—the band of ultraviolet that causes synthesis of vitamin D-3—from direct sunlight, to obtain sufficient vitamin D. Extending sunlight exposure and consuming vitamin D fortified foods may be appropriate strategies to avoid possible vitamin D deficiencies [92]. Adequate levels of this molecule and the maintenance of proper metabolism during pregnancy need to be emphasized because some beneficial effects have been reported. For example vitamin D enhances insulin responsiveness for glucose transport, through the modulation of insulin receptor expression. Vitamin D induces insulin secretion and decreases insulin resistance preventing gestational diabetes. In addition, it plays a critical role in the regulation of blood pressure and electrolyte and plasma volume homeostasis; therefore, normal serum vitamin D levels help to prevent hypertension and PE through suppression of the renin–angiotensin system [163]. Nevertheless, excess vitamin D may also have detrimental effects because it might decrease progesterone concentrations causing a preterm labor or impair fertility outcomes, so supplementation should be selective [164].

Vitamin D supplements are the usual remedy for meeting the recommended requirements (15 μg/day). Good sources of vitamin D are found in fish, liver oils, fatty fish, and egg yolks, but vitamin content in these foods varies by the time of year [63]. Plant sources of vitamin D are beans, broccoli, and leafy greens, but its content of this molecule without fortification is low, except for some wild mushrooms that contain a relevant concentration of this vitamin; therefore, vegetarian and vegan women are at risk of vitamin D deficiency. These source of vitamin D can be further fortified with calcium supplements, including cow’s milk, some soymilk products, and some breakfast cereals. Serum levels of vitamin D are easily measured if there is concern regarding intake of this essential nutrient [94]. Optimal serum 25-OH vitamin levels for pregnant women are above 75 nmol/L (30 ng/mL). Most of prenatal vitamins contain insufficient vitamin D in order to prevent infant vitamin D deficiency, so to get the recommended doses of 1000 to 2000 IU per day, are considered safe in pregnancy [157]. A recent Cochrane review underlines that supplementing pregnant women with vitamin D in a single or continued dose increases serum 25-hydroxyvitamin-D, and may reduce the risk of preeclampsia, low BW, and preterm delivery. However, when vitamin D and calcium are combined, the risk of preterm birth is increased. Further studies are needed to evaluate vitamin D supplementation and the correct dose during pregnancy [165].

#### 2.7.9. Vitamin B12

Vitamin B12 is not found in plant sources, and therefore must be obtained through animal consumption or via supplements [94]. Since vitamin B12 deficiency may appear in pregnant vegetarian women, in particular vegans, an adequate B12 status should be improved, so the use of B12 supplements is necessary. Pregnant and lactating vegans should be advised to supplement their diets with 4 µg/d of vitamin B12 [150]. Foods fortified with vitamin B12 include meat substitute products, breakfast cereals, soymilks, tofu, cereals, and nutritional yeast. Seaweed and tempeh are generally not reliable sources of vitamin B12. Four servings daily of vitamin B12-fortified foods are recommended in pregnancy and lactation [63]. Even if common pre- and postnatal multivitamins contain 100% of the RDA for vitamin B12, they are not positively associated with B12 concentration in breastmilk of vegetarian women because only a fraction of the B12 they provide is absorbed. Pregnant and lactating vegetarian and vegan mothers should be encouraged to take an individual B12, supplement and dissolve it under the tongue or chew it slowly in order to increase absorption [157]. In the case of vitamin B12 deficiency, the majority of clinical studies suggest starting with high parenteral doses of B12, after which oral treatment is continued. Considering the importance of adequate intake of vitamin B12 during pregnancy and considering that supplementation is not toxic, an ingestion of higher dose of B12 supplement (50 μg/day) would ensure correct vitamin status [92]. Vitamin B12 status (serum B12, along with homocysteine and folic acid) should be regularly checked throughout pregnancy also in women with optimal B12 levels in the first trimester, and it is necessary to adjust supplementation schemes according to the laboratory results [157].

## 3. Limitations

The present study corresponds to a descriptive review in which the majority of papers evaluated maternal dietary intake through a food frequency questionnaire. The main limitations of these questionnaires are the different food list and the possible mistakes of reporting.

Most studies analyzed in this review concluded that vegetarian and/or vegan diets exert diverse effects on pregnant outcome and nutritional profile. However, some of them describe specific populations in India or Africa with lower economic status and particular idiosyncrasy. For that, ethnicity and poverty have to be taken into account as a cause of an unbalanced diet, micronutrient deficiency, and poor pregnancy outcome. Furthermore, epidemiological and biochemical studies need to be done in Western countries to corroborate the conclusions obtained in African and/or Asian regions.

Mother undernutrition could be multifactorial, so it is important to mention the difficulties to determine the molecular mechanisms promoted by each micronutrient which may affect specifically the maternal nutritional profile.

## 4. Conclusions

Diet is one of the most significant lifestyle-related factors in determining health state and predisposing the offspring to develop several diseases. Vegetarian and vegan diets are emerging worldwide due to the evidence that plant-based dietary patterns reduce the risk of coronary heart disease, high blood pressure, type 2 diabetes, and cancer. Pregnancy is a critical window of opportunity to provide dietetic habits that are beneficial for fetal healthy. It is also an exclusive condition in which the requirements of energy and micronutrients intake increase to maintain the supply of essential nutrients for fetal development. Each stage of fetal growth is dependent on appropriate maternal nutrient transfer, so a balanced diet is essential to avoid fetal complications. The choice of vegetarian or vegan diet is always in the preconception period due to ethical reasons or poor social condition, so a well-adjusted preconception nutrition is essential for healthy pregnancy. Available data demonstrated that micronutrients insufficiency and caloric restriction are more common in developing countries, where vegetarian diets are chosen because of socioeconomic reasons. On the contrary in developed countries, the consciousness and the concern of a balanced diet is taken more into account. Generally it is difficult to verify the effects of such diets on pregnancy outcomes and to separate them from other confounding factors such as ethnicity, lifestyles or smoking.

Although more high quality evidence is needed, balanced plant-based diets rich in fibers and low in fat are considered to be protective against poor pregnancy outcomes such as PE, DG, and preterm delivery. However, these protective effects disappear if micronutrients deficiencies emerge. Moreover unbalanced dietary patterns with lack of macro- and micronutrients such as proteins, vitamin B12, vitamin D, calcium, DHA, and iron are at more risk of fetal impairment (low BW, neurological disabilities, and fetal malformations). Maternal undernutrition may potentially alter fetal growth trajectory by modifying placental weight and nutrient transfer capacity; depending on the severity of the nutritional deprivation and on the timing of depletion. Thus, plant-based diets during pregnancy and lactation require a strong awareness for a complete intake of essential key nutrients and vitamin supplements, according to international guidelines.

In addition, during breastfeeding both vegetarian and nonvegetarian mothers need caloric reserves to reach sufficient energy average. The composition of human milk changes dynamically and may vary according to many maternal factors, such as nutritional status. Nutritional supplements do not change milk composition in observational studies, also proteins concentration in human milk does not vary in relation to maternal intake of vegetal or animal proteins, but maternal body composition may be associated with the nutritional value of human milk. So maternal undernutrition, producing lack of vitamin B12, vitamin D, calcium, and DHA during lactation may lead to low vitamin content in breast milk, which can cause permanent neurological disabilities in infants or low bone mineralization. 

Finally, the current manuscript supports the evidence that maternal nutritional status is the key condition for health benefits of plant-based diets. Vegetarians and vegans are at risk of nutritional deficiencies, but if the adequate intake of nutrients is upheld, pregnancy outcomes are similar to those reported in the omnivorous population. So updated evidence highlights that well-balanced vegetarian and vegan diets should be considered safe for the mother’s health and for offspring during pregnancy and lactation. In this regard, specific dietary interventions before, during, and after gestation that are aimed at improving diet quality and adjusting appropriate intakes of macro- and micronutrients might avoid maternal health impairment, mental diseases during pregnancy, and consequent physical and neurological fetal disabilities. The vegetarian-type pattern should be considered safe and it is not associated with preterm birth, BW, or SGA if the requirements are met. Therefore, healthcare professionals might have knowledge about plant-based diets characteristics in order to implement balanced dietary patterns, enhancing supplement intake, and paying attention to critical nutrients to avoid dangerous health outcomes. Further large-scale observational studies would help to define correlations between plant-based diets, gestation, and health, and might be suitable to design pregestational nutrition intervention strategies.

## Supplementary Materials

The following are available online at https://www.mdpi.com/2072-6643/11/3/557/s1, Table S1: Selected daily nutrient requirements during pre-pregnancy, pregnancy, and lactation; Table S2: Studies about the effects of vegetarian and vegan diet on maternal nutritional profile; Table S3. Studies about the effects of Vegetarian and vegan diet on maternal outcomes; Table S4: Studies about the effects of vegetarian and vegan diets on fetal outcomes.
