# Supplementary material for: The Effects of Vegetarian and Vegan Diet during Pregnancy on the Health of Mothers and Offspring
Source: Nutrients. 2019 Mar 6;11(3):557. doi: 10.3390/nu11030557 (PMC6470702; doi:10.3390/nu11030557)
Supplement: Supplementary file 1 [file nutrients-11-00557-s001.pdf]

Table S1. Selected daily nutrient requirements during pre-pregnancy, pregnancy and lactation

	Pre-pregnancy	Pregnancy				Lactation		
**Nutrient**	**RCOG/IOM**	**RDA american**	**DRI/IOM**	**EFSA**	**FIGO**	**DRI/IOM**	**EFSA**	**FIGO**
Energy (kcal/d)	1940	2099 +340 since 2nd trim.	+340 2nd trim, +452 3rd trim	+ 70 1st trim, +260 2nd trim. +500 3rd trim.	+ 69 1st trim, +266 2nd trim, + 496 3rd trim.	+500	+500	+500
Protein (g/d)	45 (0.8g/kg/d)	71(1.2g/kg/day)	60 (1.1g/kg/d)	+ 26	71	+ 25	+ 15	a
Carbohydrate (g/d)	130	175	175	175	175	210	210	210
Thiamin (mg/d)	0.8/1.1	1.4	1.4	0.8	a	1.4	0.1	a
Riboflavin (mg/d)	1.1	1.4	1.4	1.5	a	1.6	2	a
Vitamin C (mg/d)	40/ 75	85	85	85	105	120	155	a
Vitamin E (mg/d)	15	15	15	11	15	19	19	a
Folate (μg/d)	200/ 400	600	600-800	600	600	500	500	600
Vitamin D (μg/d)	5	15	+ 10	15	15	15	15	15
Vitamin A (μg/d)	600/700	770	770	700	750-770	1300	1300	1300
Niacin (mg/d)	13/ 14	18	18	16	a	17	17	a
Vitamin B6 (g/d)	1.2/ 1.3	1.9	1.9	1.5	1.9	2	1.7	2
Vitamin B12 (μg/d)	2.4	2.6	2.6	4.5	2.6	2.8	5	2.8
Vitamin K (mg/d)	90	-	90	70	-	90	70	-
Calcium (mg/d)	700/ 1000	1000	1000	1000	1000-1300	1000	1000	1000-1300

Magnesium (mg/d)	270	350	a	300	a	300	300	a
Sodium (mg/d)	1600	1500	a	a	a	a	a	a
Chloride (mg/d)	2500	2500	a	a	a	a	a	a
Potassium (mg/d)	3500	4700	a	3500	a	a	4000	a
Iron (mg/d)	15/ 18	27	27	27-30	27	9	11-20	9
Zinc (mg/d)	7/ 8	11	11	+ 1.6	11-12	12	+ 2.9	12
Copper (mg/d)	1.2	1	1	1.5	1	a	1.5	1
Selenium (µg/d)	60 / 55	60	60	70	60	70	85	70
Iodine (µg/d)	140	200	200	200	220	250	200	290
DHA(mg/d)/ Ω3(g)	250/ 2	+ 200 / 1.4		+100-200	+200/ 1.4	+ 100-200	+100-200	+ 200/ 1.3
Phosphorus (mg/d)	550/ 700	700	700	550	550	700	550-700	a

a= no increment during pregnancy or lactation

Table S2. Studies about the effects of Vegetarian and Vegan diet on maternal nutritional profile					
**Author (year)**	**Refer.**	**Aim of Study**	**Type of study / Population**	**Maternal nutritional profile**	**Key Results**
Sanders (1999)	[87]	To review the evidence concerning the essential fatty acid status of vegetarians during pregnancy, lactation, and infancy and makes some recommendations with regard to dietary intake.	Narrative review	There are differences in the essential fatty acid status of vegetarians and omnivores, the most notable being the higher intakes of LA by vegetarians and, the differences in intake of LCPUFAs.	There is a lower proportion of DHA in the blood and tissue lipids of vegans and vegetarians
Koebnick (2001)	[71]	To compare folate status during pregnancy in vegetarian pregnant women and in consumers of WD	Longitudinal case-control n=109	Long-term high vegetable intake favourably affects plasma and Red Blood Cells (RBC) folate concentrations during pregnancy and reduces the risk of folate deficiency with adequate vitamin B12 supply	-Folate deficiency: OR 0.1 (95% CI 0.01–0.56) in LOV, OR 0.52 (95% CI 0.2–1.34) in LME versus WD.<br>-RBC folate concentrations in LOV was positively associated to vitamin B12 intake ($r = 0.51$, $P < 0.0001$)
Sharma (2003)	[83]	To assess the effect of dietary habits on prevalence of anemia in pregnant women of Delhi	Cross sectional n=1150	Very high prevalence of anaemia during pregnancy, no difference according to diet	Anaemia (%): 96.18 (vegetarians), 95.3 (halal meat eaters), 96.2 (jhatka meat eaters), (ns)
Koebnick (2004)	[67]	To compare serum vitamin B12 and homocysteine concentrations in pregnant women consuming either a LOV diet, a low meat diet (LMD) (<300 g/wk), or a diet with larger amounts of meat (>300 g/wk)	Longitudinal Cohort n=109	Pregnant women consuming a predominantly vegetarian diet had an increased risk of vitamin B12 deficiency	-Vitamin B12 intake (lg/day): 2.5 (1.3–3.8) in LOV, 3.8 (3.0–4.9) in LMD, 5.3 (4.3–6.3) in controls ($P < 0.001$).<br>-Lower serum vitamin B12 levels in LOV ($P < 0.001$) and LMD ($P = 0.05$) versus controls.<br>-Higher plasma total homocysteine in

					LOV (P = 0.032) and LMD (P = 0.061) versus controls
Koebnick (2005)	[92]	To explore the relationship between habitual vegetarian diets and dietary zinc intake/status during pregnancy.	Longitudinal cohort<br>Total n =108	Improved magnesium status and lower frequency of calf cramps during pregnancy in plant-based diets	-Dietary magnesium intake (mg/day): 508 ±14 (LOV), 504 ± 11 (LME), 412± 9 (controls) (P < 0.001).<br>-No significant difference in serum and RBC magnesium between groups.<br>-Urinary magnesium excretion higher in LOV (P = 0.023) and LME (P = 0.017) versus controls.<br>-Lower occurrence of calf cramps in LOV (P = 0.004) and LME (P = 0.008)
Sachan (2005)	[74]	To determine the prevalence of osteomalacia and hypovitaminosis D in pregnant Indian women and in cord blood and to correlate maternal 25-hydroxyvitamin D [25(OH)D] status with sun exposure, daily calcium intake (dietary plus supplemental), and intact parathyroid hormone (PTH) concentrations	Cross sectional<br>n=207	High prevalence of physiologically significant hypovitaminosis D among pregnant women especially those who didn't consume meat and their newborns	-Mean maternal serum 25(OH)D was 14 ±9.3 ng/mL, and cord blood 25(OH)D was 8.4±5.7 ng/mL. PTH was in the normal range when 25(OH)D was <22.5 ng/mL<br>-84.3% of urban and 83.6% of rural women had 25(OH)D values below that cutoff<br>-Calcium intake was low, but higher in urban than in rural subjects (p<0.001)

Gibson (2008)	[68]	To investigate the vitamin B-12 and folate status of pregnant women from Ethiopia	Cross sectional n=99	These pregnant women had early functional deficiency of vitamin B-12 but not folate deficiency and no vitamin B-12 deficiency anemia, despite very low intakes of animal products	-29% had anemia, 13% had iron deficiency anemia, 33% had depleted iron stores, 74 and 27% had low plasma zinc and retinol, respectively.<br>-Only 2% had low plasma folate (<6.8 nmol/L) and 23% had low plasma vitamin B12 (<150 pmol/L), even though 62% had elevated plasma MMA (> 271 nmol/L); p< 0.05. None had elevated plasma cystathionine or total homocysteine
Kazemi (2009)	[75]	To determine the prevalence of hypovitaminosis D in pregnant Iranian women and to examine the probable correlation between serum concentrations of 25(OH)D in maternal blood and in neonates' cord blood	Cross sectional n=67	High prevalence of physiologically significant hypovitaminosis D among Iranian pregnant women and their newborns,	-Mean maternal serum 25(OH)D was 19.4± 3.9 nmol/L, and cord blood 25(OH)D was 16.7 ± 2.9 nmol/L.<br>- During winter 86% of the women had Hypovitaminosis D and 75% of the newborns; during summer 46% of the mothers and 35% of the newborns<br>-A positive correlation was found between maternal and cord blood 25(OH)D (r = 0.55, p < 0.001).
Alwan (2011)	[82]	To investigate the association between iron intake in pregnancy and size at birth in UK	Prospective cohort n=1274	Iron intake, both from diet and supplements, during the first trimester of pregnancy was higher in vegetarians and women with a better socioeconomic profile	-Women with dietary iron intake >14.8 mg/day were older, have a higher socioeconomic profile and take supplements during the first trimester.<br>-Vegetarians were less likely to have low dietary iron intake [OR = 0.5, 95% ; CI: 0.4,

					0.8] and more likely to take supplements<br>-Total iron intake, was associated with birthweight centile (adjusted change = 2.5 centiles/10 mg increase in iron, 95% CI: 0.4, 4.6).
Dasgupta (2012)	[76]	To look for any association of vitamin D status during pregnancy with the modifiable factors - extent of sun exposure, sunscreen use, vegetarian diet, dietary calcium intake, and multivitamin supplementation in North Eastern part of India	Case-control<br>n=50	Sun exposure, sunscreen use, and vegetarian diet are important modifiable variables significantly associated with vitamin D status in pregnancy.	-42% of the cases were found to have vitamin D deficiency and 14% vitamin D insufficiency, 63.63% of whom were vegetarian (P =0.0375).<br>-There was a significant association of 25(OH)D levels with extent of sun exposure, sunscreen use, and vegetarian diet.<br>-There was no association of 25(OH)D levels with multivitamin supplementation or dietary calcium intake.
Simões-Wüst (2013)	[61]	To describe maternal BMI according to dietary pattern (vegetarian vs conventional)	Longitudinal cohort<br>n=2818	Vegetarian lifestyle is associated with low body weight lower prevalence of overweight and obesity than the conventional group, before pregnancy and 4-5 years after delivery	Mothers of vegetarian group showed:<br>-lower BMI (22.6 *vs*.23.9 kg/m^2, $P < 0.001$)<br>- lower blood pressure (2.5vs5%,p= 0.015),<br>-more candida infections (23 vs 14.2 %, p< 0.01)<br>- lower BMI 4-5 years after delivery (74.7 *vs*. 65.3%, p<0.001)

Gadgil (2014)	[70]	To evaluate if plasma maternal folate, vitamin B-12 and homocysteine levels had an effect on maternal global DNA methylation in Indian pregnant women	Cross sectional n= 49	Moderate vitamin B-12 deficiency in vegetarian pregnant women might be the<br>cause of hyperhomocystinemia and hypermethylation when compared to vitamin B-12 sufficient non-vegetarian group.	-Folate and vitamin B-12 were inversely correlated to homocysteine in pregnant vegetarian women and non-vegetarian<br>-No difference in global DNA methylation was found between the vegetarian and non-vegetarian<br>-Folate and vitamin B-12 did not show association with global DNA methylation,<br>-Plasma total homocysteine of the vegetarian group showed significant correlation to global DNA methylation (**r**2**.**0.49,**p.**0.011).
Foster (2015)	[91]	To assess the zinc status of vegetarians during pregnancy compared to non-vegetarians (NV)	Meta-analysis	Pregnant vegetarian women have lower zinc intakes than NV control populations and both groups consume lower than recommended amounts.	-Zinc intake of vegetarians was lower than that of NV (−1.38 ± 0.35 mg/day; $p < 0.001$)<br>-The exclusion of low meat eaters from the analysis revealed a greater difference (−1.53 ± 0.44 mg/day; $p = 0.001$).<br>-Neither vegetarian nor NV groups met the RDA for zinc.<br>-There is no difference between groups in biomarkers of zinc status or in functional outcomes associated with pregnancy
Jeruszka-Bielak (2017)	[69]	To determine B12 status and the prevalence of B12 deficiency, using combined plasma total B12 and MMA concentrations in a sample of healthy	Cross sectional n=320	South Asian pregnant women were at particular risk for B12 deficiency, due to lower animal source food intake	-Plasma total B12 concentrations indicative of deficiency (<148 pmol/L) and suboptimal B12 status (148–220 pmol/L) were found in 18% and 33% of the

		pregnant women in Metro Vancouver			women, respectively.(p<0.01)<br>-Normal plasma MMA concentration (<210 nmol/L) was observed in 82%of all women.<br>-Gestational age was a strong predictor of plasma B12 and MMA concentration, and South Asian ethnicity of B12 deficiency and MMA concentrations (p<0.01)<br>-BMI was strong predictor of total B12 (p<0.01)
Simões-Wüst (2017)	[60]	To determine consumer characteristics, dietary patterns and macro- and micronutrient intakes associated with consumption of organic food during pregnancy	Longitudinal Cohort n=2786	Consumption of organic food is associated with food patterns comprising more vegetable products and fewer animal products with balanced intake of macro and micro nutrients	Distinct intakes of macro- and micronutrients, including higher retinol, carotene, tocopherol and folate intakes, lower intakes of vitamin D and B12 and specific types of trans-fatty acids was found in the organic groups (p<0.01)
Young (2017)	[78]	To describe nutrient intakes, characterize dietary patterns and analyse their associations with sociodemographic characteristics among pregnant women in Shaanxi, China	Population-based cross-sectional n=4762	Women with high scores on the vegetarian pattern and snacks pattern tended to be in low balanced pattern score groups, and had lower nutrient intakes than those in the high balanced pattern score groups	Pregnant women in Shaanxi, China had low intakes of most nutrients such as vitamin A, folate and Calcium. Dietary patterns and most nutrient intakes varied by sociodemographic characteristics (p<0.001)
Elsori (2018)	[77]	A systematic approach has been developed for the assessment of data in regards of vitamin D deficiency among mothers, neonates, and children.	Systematic review	Vitamin D deficiency mainly occurs if strict vegetarian diet is followed as mostly the source of vitamin D is animal based; therefore, exposure to sunlight is restricted or having dark skin color.	Vitamin D deficiency is common among pregnant women especially vegetarians, women from developing countries and dark skin color.

Piccoli (2015)	[127]	To review the literature on vegan–vegetarian diets and pregnancy outcomes.	Narrative Review	Vegan–vegetarian diets may be considered safe in pregnancy, provided that attention is paid to vitamin and trace element requirements.	-The duration of pregnancy was available in six studies and was similar between vegan–vegetarians and omnivores.<br>-The nine heterogeneous studies on microelements and vitamins suggest vegan–vegetarian women may be at risk of vitamin B12 and iron deficiencies

Table S3. Studies about the effects of Vegetarian and Vegan diet on maternal outcomes					
**Author (year)**	**Refer.**	**Aim of Study**	**Type of study / Population**	**Maternal outcomes**	**Key Results**
Carter (1997)	[95]	To examine the maternity care records vegan mothers for symptoms of preeclampsia	Observational retrospective n=775	Vegan diet might be protective against PE	-Only a case of preeclampsia (0.13%).<br>-PE may be caused by a relative prostacyclin deficiency secondary to an excessive production of thromboxane A2.<br>-A vegan diet (low in AA) might provide protection against this condition,
Olsen (2002)	[110]	To assess if low consumption of seafood in early pregnancy is a risk factor for preterm delivery in Denmark	Prospective cohort n=8729	Low consumption of seafood was a risk factor for preterm delivery	Low consumption of seafood was a strong risk factor for preterm delivery and low birth weight, OR=3.6 (95% confidence interval 1.2 to 11.2)
Khoury (2005)	[109]	To determine the effect of a cholesterol lowering diet on maternal, cord, and neonatal lipids, and pregnancy outcome (Norway)	Randomized clinical trial n=290	-A cholesterol-lowering diet may modify maternal lipid levels but not cord and neonatal lipids.<br>-Marked observed effect of this diet on the reduction of preterm delivery in low-risk pregnancies	-Maternal total and LDL cholesterol levels were lowered in the intervention group (p<0.01)<br>-Lipid levels in cord blood and in neonates born to mothers in the intervention versus the control groups did not differ.<br>-Preterm birth< 37 weeks was lower in the intervention group, (RR 0.10; 95% CI 0.01-0.77) in low risk pregnancies.

Frederick (2005)	[98]	To explore the relation between preeclampsia risk and maternal intake of dietary fiber, potassium, magnesium and calcium.	Case- control n=511	Diets rich in fiber and potassium are associated with a reduced risk of hypertension	Comparing extreme quartiles of total fiber intake, the OR for PE was 0.46 (95% CI: 0.23-0.92). The multivariate OR for PE for women in the top quartile of potassium intake (> 4.1 g/d) versus the lowest quartile (< 2.4 g/d) was 0.49 (95% CI 0.24-0.99).<br>-Intake of fruits and vegetables, low-fat dairy products, total cereal and dark bread were each associated with a reduced risk of preeclampsia.
Zhang (2006)	[99]	To examine whether pregravid dietary fiber consumptions from cereal, fruit, and vegetable sources and dietary glycemic load were related to GD risk. Nurses' Health Study II (USA)	Observational Prospective n=13110	Diet with low fiber and high glycemic load was associated with an increased risk.	-758 cases of GD in an 8 years period<br>-Each 10-g/day increment in fiber intake was associated with 26% (95% CI 9–49) reduction in GD risk.<br>-Each 5g/day increment in cereals with a 23% (9–36) risk reduction and or fruit fiber with a 26% (5–42) risk reduction of GD<br>-Combination of high–glycemic load and low–cereal fiber diet was associated with 2.15-fold (1.04–4.29) higher risk of GD
Longo-Mbenza (2008)	[97]	To assess the frequency pf pregnancy-induced hypertension and if vegetables intake and physical activity are protective against pregnancy-induced hypertension onset among rural women from Democratic republic of	Longitudinal n= 238	Diet rich in vegetables, overweight reduction and physical activity among pregnants prevent pregnancy-induced hypertension.	-The onset of PE was higher within vendors and inactive women as well as among vegetarians (3.1%) than women with diet high in meat (9.7%).<br>-PE occurred more ($p<0.05$) among

		Congo.			pregnants with rare daily servings of vegetables (33.3%) than pregnants with 3 or more (3.7%).<br>-Physical activity (RR=0.63 CI 95% 0.33 to 0.94) and >3 daily servings of vegetables (RR=8.8 CI 95% 0.6 to 0.98) were protective factors against PE (p<0.01)
Qiu (2008)	[96]	To assess dietary fiber intake in early pregnancy and risk of subsequent preeclampsia in Washington state	Prospective n=1.538	Dietary fibers reduce dyslipidaemia, condition related to PE.	-Fiber intake: RR of preeclampsia for women in the highest (≥21.2 g/day) vs. the lowest quartile (<11.9 g/day) was 0.28 (95% CI = 0.11–0.75). Also, for water-soluble fiber and insoluble fiber.
Mikkelsen (2008)	[107]	To examine if maternal intake of MD is associated with reduced risk of preterm birth Danish National Birth Cohort non-smoking women	Prospective cohort n=35530	Shifting towards a MD during pregnancy may reduce the risk of early delivery in Danish women.	OR for preterm birth and early preterm birth were 0.61 (95% Confidence Interval (CI): 0.35-1.05) and 0.28 (0.11-0.76), respectively, in MD women compared to women fulfilling none of the MD criteria.
Streuling (2011)	[105]	To evidence on whether diets with lower caloric/protein content or other diets might be associated with lower GWG	Systematic review	GWG might be reduced by lower energy intake in pregnancy as in vegetarian diets	12 studies<br>-5 studies suggested significant positive associations between energy intake and GWG, 3 found no significant association.<br>-Further significant positive associations of GWG were reported with respect to protein intake, animal lipids, energy density and a number of different food servings per day

					-Intake of carbohydrates and vegetarian diet were associated with less GWG.
Hogg-Kollars (2011)	[114]	To assess the relationship of consumption-frequency of foods and supplements rich in nutrients and post-partum depression	Retrospective Survey	Depression was more prevalent among women with vegetarian diets	-Depressed subjects consumed oily fish and offal significantly more often than non depressed subjects.<br>-Depression was more prevalent among women with vegetarian diets.<br>-The majority of subjects with selfreported depression described nutritional support during pregnancy as inadequate.
Robic (2012)	[125]	To compare Body composition of vegetarian and nonvegetarian pregnant women	Longitudinal cohort n=27	BMI was lower in vegetarian than nonvegetarian with the same body height but did not affect birth mass of neonates	-BMI 32 weeks of gestation: 23.2 ±1.8 (vegetarians), 24.3 ± 3.2 (non-vegetarians) ($P < 0.05$).<br>-No differences in body mass, BMI, body fat percentage, or pregnancy weight gain at birth and 6 weeks after delivery
Wen (2013)	[119]	To assess the associations of diet and physical activity with the three components of gestational weight gain	Cohort n=852	No association between vegetarian diet during second trimester and maternal weight gain	No association between vegetarian diet during second trimester and maternal weight gain
Grieger (2014)	[106]	To identify associations between maternal dietary patterns in the 12 mo before conception on fetal growth and preterm delivery in Australia	Retrospective n=309	A dietary pattern containing several protein-rich food sources, fruit, and some whole grains is associated with reduced likelihood for preterm delivery,	-A 1-SD increase in the scores on the high-protein/fruit pattern was associated with decreased of preterm birth (OR: 0.31; 95% CI: 0.13, 0.72; $P = 0.007$)<br>-The reverse direction was apparent for the high-fat/sugar/takeaway pattern (OR:

					1.54; 95% CI: 1.10, 2.15; P= 0.011).<br>-A 1-SD increase in the scores on the high fat/sugar/takeaway pattern was also associated with shorter gestation (regression coefficient: 22.7; 95% CI: 24.3, 21.1; P= 0.001) and birth length (adjusted regression coefficient: 20.5; 95% CI: 20.8, 20.1; P = 0.004).
Asemi (2014)	[100]	To investigate the effects of DASH diet on pregnancy outcomes in Iranian women with gestational diabetes	Randomized controlled clinical trial (RCT) n=52	Consumption of DASH diet for 4 weeks among pregnant women with GD resulted in improved pregnancy outcomes.	-Cesarean section: 46.2% vs 80.8% control group (P<0.01).<br>-Need for insulin after intervention: 23% vs 73% for control group, (p<0.0001).
Arora (2015)	[102]	To determine the prevalence and risk factors of GD comparing the previous World Health Organization (WHO) 1999 criteria to the WHO 2013 criteria in North India.	Cross sectional n=5100	With the WHO 2013 GD criteria the prevalence of GD increased in North Indian pregnant women.	-The prevalence of GD was 35% using WHO 2013 criteria vs 9% using WHO 1999 criteria.<br>-Fasting plasma glucose (FPG) measurements identified 94% of WHO 2013 GD cases as opposed to 11% of WHO 1999 GD cases
Knight (2015)	[103]	To explore the relationship of circulating serum vitamin B12 and folate at 28 weeks' gestation with maternal adiposity and related biochemical markers in a white non diabetic UK obstetric cohort.	Cross-sectional n=995	Associations between lower serum B12 higher obesity and insulin resistance during pregnancy in a non- diabetic White British population.	-HIgher BMI was associated with lower circulating vitamin B12 (r = -0.25; P<0.001) and folate (r = -0.15; P<0.001).<br>-In multiple regression analysis higher BMIwas an independent predictor of lower circulating B12 (β (95% CI) = -0.59 (-0.74, -0.44)

					-Other markers of adiposity/body fat metabolism (HOMA-R, triglycerides and AST) were also independently associated with circulating B12.<br>-In a similar multiple regression AST was the only independent obesity-related marker associated with serum folate (β (95% CI) = 0.16 (0.21, 0.51)).
Muktabhant (2017)	[104]	To determine dietary advice interventions in pregnancy for preventing gestational diabetes mellitus	Review Cochrane<br>49 RCT<br>n=11400	More high-quality evidence is needed to determine the effects of dietary advice interventions in pregnancy.	Very low-quality evidence from trials suggests a possible reduction in GD risk for women receiving dietary advice versus standard care, and low-quality evidence from trials suggests no clear difference for women receiving low- versus moderate- to high-GI dietary advice
Olsen (2018)	[111]	To examine the association between plasma fatty acids quantified in pregnancy and subsequent risk of early preterm birth	Prospective cohort	Low plasma concentration of EPA and DHA during pregnancy is a strong risk factor for subsequent early preterm birth in Danish women	-Women in the lowest quintile (EPA+ DHA $< 1.6\%$) had 10.27 times (95% confidence interval 6.80-15.79, $p < 0.0001$) increased risk of preterm birth<br>-Women in the second lowest quintile had 2.86 (95% CI 1.79-4.59, $p < 0.0001$) times increased risk, when compared to women in the three aggregated highest quintiles (EPA+DHA $\geq 1.8\%$).

Assaf-Balut (2019)	[101]	To evaluate the effect of (MedDiet), enhanced with extra virgin olive oil (EVOO) and nuts, on a composite of adverse maternofoetal outcomes of women with normoglycemia during pregnancy.	Randomized controlled clinical trial n=697	A MedDiet, enhanced with EVOO and nuts, was associated with a risk reduction of maternofoetal outcomes over 50% in normoglycemic pregnant women	-The authors found a significant reduction in the risk of composite outcome (OR= 0.48 [0.37–0.63]; p = 0.0001), NNT = 5. -No differences in physical activity among groups.

Table S4. Studies about the effects of vegetarian and vegan diets on fetal outcomes.					
**Author (year)**	**Refer.**	**Aim of Study**	**Type of study**	**Fetal Outcomes**	**Key Results**
Reddy (1994)	[120]	To determine fatty acid composition of plasma phospholipids of cord blood in 24 Asian vegetarian and 24 white omnivore non-pregnant premenopausal women	Prospective Study n=144	Birth weight, head circumference and length were lower in the infants born to South Asian vegetarians	-The proportion of DHA was lower (P > 0.001) but that of docosapentaenoic acid (22:5n-6) was greater (P < 0.001) in cord blood from the omnivores<br>-On vegetarian women early onset of labour and shorter duration of gestation was detected
North (2000)	[134]	To determine the role of vegetarian diet and intake of phytoestrogens during pregnancy and the incidence of hypospadias	Prospective study n=7928	Hypospadias	Vegetarian pregnants had an OR of 4.99 (95%, CI, 2.10±11.88) of hypospadias in their newborns. No significant differences in the incidence of hypospadias were found related to intake of soya milk.
Phillips (2005)	[9]	To investigate the outcomes of vegetarian nutrition in pregnancy	Briefing paper	Consuming a vegetarian diet during pregnancy was associated with an increased risk of giving birth to a boy with hypospadias	-The lower birth weight observed among infants born to mothers on vegetarian diets may be related to poor nutritional status with regard to iron, or folate and/or vitamin B12
Giordano (2007)	[138]	To assess the a relationship between hypospadias and cryptorquidism and maternal diet	Case-control Prospective study n= 292	Hypospadias and Cryptorquidism	Increased ORs of hypospadias in pregnants who frequently consumed fish (OR = 2.33 [95% CI 1.03, 5.31]) and market-purchased fruit (OR = 5.10 [95% CI 1.31, 19.82]). For cryptorchidism, increased risk was observed in mothers consuming liver (OR = 5.21 [95% CI 1.26, 21.50]), and smoked products (OR = 2.46 [95% CI 1.15, 5.29]).

Akre<br>(2008)	[135]	To investigate the relationship between hypospadias and exposure to exogenous hormones and maternal diet during pregnancy.	Case-control<br>Prospective study<br>n=719	Hypospadias	The lack of meat and fish in pregnant diet appears to increase the risk of hypospadias with an OR of 4.6 (95% CI, 1.6–13.3]
Ramón<br>(2009)	[122]	To examine the relationship between consumption of fruit and vegetables during pregnancy and anthropometric measures at birth in a general population mother-infant cohort (Spain)	Case-control<br>n=787	Vegetable consumption throughout pregnancy may have a beneficial effect on fetal growth.	Fruits and vegetables categorized in quintiles.<br>-Women with less intake during the first trimester had higher SGA (OR, 3.7; 95% CI: 1.5-8.9; p-trend < 0.001)<br>-No association between fruit consumption and birth outcomes.
Li<br>(2011)	[141]	To examin the association between maternal periconceptional consumption of pickled vegetables and NTDs in Shanxi Province of northern China.	Case-control<br>n=1213	Maternal periconceptional consumption of pickled vegetables may increase the risk for NTDs in Shanxi Province	-The risk of NTDs was positively associated with the consumption of pickled vegetables by the mother.<br>-Compared with pickled vegetables consumption at < 1 meal/week, the adjusted OR for consumption at 1 - 3, 4 - 6, and > 6 meals/week were 1.3 (95% CI: 1.0, 1.8), 1.9 (1.1, 3.2), and 3.6 (1.9, 6.9), respectively.<br>-A protective effect was found for maternal meat consumption at ≥ 1 meal/week (adjusted OR: 0.6, 95% CI:

					0.4, 0.7) and egg or milk consumption at ≥ 1 meal/week (adjusted OR: 0.6, 95%CI: 0.4, 0.8).
Deb (2011)	[131]	To analyze the potential role of MTHFR gene polymorphism, folate supplementation and dietary pattern among the mothers of NTD neonates and controls in heterogeneous populations of North India	Case-control n=333	Significant increased risk for NTD with respect to low folic acid supplementation and vegetarian diet in univariate and multivariate analyses.	-No significant difference in the genotypic or allelic distribution of MTHFR C677T polymorphism, however, high frequency of CT genotype among controls suggests heterozygous advantage probably due to supplementary folate.<br><br>-Among the two communities, Muslim NTD mothers had higher TT genotype showing increased risk for NTD (adjusted OR: 12.9; 95% CI: 1.21-136.8) and lower folic acid supplementation (adjusted OR: 3.5; 95% CI: 1.18-10.22). Whereas, marginal increased risk for NTDs with vegetarian diet was observed among Hindus
De kort (2011)	[140]	To asses the role of maternal diet patterns in the incidence of hypospadias	Case-control Prospective study n=961	Hypospadias	Low frequency of consumption of yoghurt, cheese, eggs, fruit and vegetables, fish, beans and pulses, olive oil and organic food had a higher risk of hypospadias (OR 1.54; 95% CI 1.06, 2.26) compared with high frequency of consumption of fresh fruit and vegetables, dried fruit, fresh or frozen

					fish, beans, pulses, soya products, olive oil and organic food
Robic (2012)	[125]	To compare Body composition of vegetarian and nonvegetarian pregnant women and the effect on neonate BW	Abstract	No significant differences between groups in pregnancy weight gain and birthweight were observed	No differences in body mass, body fat percentage, or pregnancy weight gain at birth and 6 weeks after delivery were detected between vegetarians and omnivorous women
Carmichael (2012)	[137]	To investigate the relationship of hypospadias and maternal intake of animal products, vegetarian diet and estrogen metabolism.	Prospective Study n=4368	Hypospadias	Intake of animal products was not associated with hypospadias OR for any versus no intake of meat was 1.0 (95% CI 0.6, 1.6). Frequency of intake of meat or other animal products was also not associated with hypospadias, nor was intake of iron or several nutrients that are potentially related to estrogen metabolism
Wen (2013)	[119]	To assess associations of diet and physical activity with the three components of GWG	Prospective n=2128	Healthful diet and greater physical activity are associated with reduced risk for excessive gestational weight gain.	Vegetarian diet during second trimester associated with lower fetal [-0.39 (-0.71, -0.08)] and placental weight [-0.40 (-0.79, -0.01)]
Carmichael (2013)	[136]	To determine the role of phytoestrogens in maternal diet and the risk of hypospadias	Prospective n=4368	Hypospadias	OR of hypospadias for total phytoestrogen intake was 0.7 (95% CI: 0.5, 1.0)

Haider (2013)	[132]	To investigate the associations of maternal anaemia and prenatal iron use with maternal haematological and adverse pregnancy outcomes; and to evaluate potential exposure-response relations of dose of iron, duration of use, and haemoglobin concentration in prenatal period with pregnancy outcomes	Systematic review and meta-analysis: 48 randomised trials and 44 cohort studies	Daily prenatal use of iron improved BW probably leading to a reduction in risk of low BW. An improvement in prenatal mean haemoglobin concentration linearly increased BW.	-Iron use increased maternal mean haemoglobin concentration by 4.59 (95% CI 3.72 to 5.46) g/L and reduced the risk of anaemia (RR 0.50, 0.42 to 0.59), iron deficiency (0.59, 0.46 to 0.79), iron deficiency anaemia (0.40, 0.26 to 0.60), and low BW (0.81, 0.71 to 0.93).<br><br>-The effect of iron on preterm birth was not significant (relative risk 0.84, 0.68 to 1.03). -Analysis of cohort studies showed a significantly higher risk of low BW (adjusted OR 1.29, 1.09 to 1.53) and preterm birth (1.21, 1.13 to 1.30) with anaemia in the first or second trimester. -Birth weight increased by 15.1 (6.0 to 24.2) g (P for linear trend=0.005) and risk of low BW t decreased by 3% (relative risk 0.97, 0.95 to 0.98) for every 10 mg increase in dose/day (P for linear trend<0.001).<br><br>- Duration of use was not significantly associated with the outcomes after adjustment for dose. For each 1 g/L increase in mean haemoglobin, birth weight increased by 14.0 (6.8 to 21.8) g (P for linear trend=0.002);

					-No evidence of a significant effect on duration of gestation, SGA births, and birth length was noted.
Fikawati (2013)	[124]	To examine nutrient intake of vegetarian pregnant mothers in Indonesia in the relation to the outcome of pregnancy	Prospective<br>n= 85	Vitamin B12 was utilized mainly for fetal development rather than maternal tissue development so there is a negative impact of vitamin B12 deficiency on fetus	-Macronutrients intake among vegetarian mothers were higher than RDA, but the intakes of micronutrients were lower than the RDA. Intake of these nutrients (except vitamin B12 intake) was associated significantly with pregnancy weight gain.<br>-Intake of vitamin B12 was significantly associated with infant BW.
Christensen (2013)	[139]	To examine the association between organic dietary choice during pregnancy and presence of hypospadias in the offspring.	Case-control<br>n=306		-Organic choice of food items during pregnancy was not associated with hypospadias in the offspring.<br>-However, frequent current consumption of high fat dairy products (milk, butter) while rarely or never choosing the organic alternative to these products during pregnancy was associated with increased odds of hypospadias (adjusted OR 2.18, 95% CI 1.09-4.36).
Larsen (2014)	[130]	To examine the association between maternal vegetarianism and the risk of	Cohort<br>n=80.743	A large and wealthy population with ample access to healthy food and dietary	The number of children with a diagnosis of impaired neurodevelopment among the

		impaired neurodevelopment of Danish National Birth Cohort		supplements do not report impaired neurodevelopment in children of vegetarian mothers.	lactoovo-vegetarian and vegan groups was too low to perform statistical tests.
Kjøllesdal (2014)	[118]	To review studies investigating the associations between dietary patterns derived from *a posteriori* analysis and BW, or being SGA in New Zealand	Review	Dietary patterns associated with lower BW or higher risk for giving birth to a SGA baby were characterized by high intakes of processed and high fat meat products, sugar, confectionaries, sweets, soft drinks, and unspecified or refined grains.	Dietary patterns positively associated with BW were labeled "nutrient dense", "protein rich", "health conscious", and "Mediterranean". Those negatively associated with BW were labeled "Western", "processed", "vegetarian", "transitional", and "wheat products". The dietary patterns "Western" and "wheat products" were also associated with higher risk of SGA babies, whereas a "traditional" pattern was inversely associated with SGA baby.
Murphy (2014)	[121]	To assess the associations of consumption of fruits and vegetables during pregnancy with infant BW and SGA	Systematic review	Limited evidence of a positive association between fruit and vegetable consumption during pregnancy and BW	-One prospective study from a highly developed area reported increased risk for SGA by women with low vegetable intakes (odds ratio 3.1; 95% confidence interval 1.4-6.9; P=0.01);<br>-another large prospective study reported a 10.4 g increase in BW per quintile increase in fruit intake (95% confidence interval 6.9-3.9; P<0.0001)

					and increases of 8.4 or 7.7 g per quintile intake of fruits and vegetables (combined) or fruits, vegetables, and juice (combined), respectively.<br>-One retrospective study reported an association between low fruit intake and BW. In less developed countries, increased vegetable or fruit intake was associated with increased BW in two prospective studies.
Fikawati (2014)	[147]	To analysis the association between predominant breastfeeding and the nutritional status of lactating vegetarian mothers and growth of their breastfed infant	Prospective study	There was no difference in birth weight and growth curves between infants of vegetarian and nonvegetarian mothers	Infants may be born with low vitamin B12 stores if the maternal intake during pregnancy is inadequate
Piccoli (2015)	[127]	To review the literature on vegan–vegetarian diets and pregnancy outcomes.	Review	There are no evidence of adverse fetal outcomes in infants from vegetarian or vegan mothers	-None of the studies reported an increase in severe adverse outcomes or in major malformations, except one report of increased hypospadias in infants of vegetarian mothers.<br>-Five studies reported vegetarian mothers had lower BW babies, yet two studies reported higher birthweights.

Zulnyak (2017)	[119]	To investigate significant variation in diet during pregnancy between ethnic groups in high-income countries on BW	Prospective n=3997	Maternal consumption of a plant-based diet during pregnancy is associated with BW. Among white Europeans, a plant-based diet is associated with lower BW, reduced odds of an infant born LGA and increased odds of SGA, whereas among South Asians living in Canada, a plant-based diet is associated with increased BW	-Among white europeans: plant-based dietary pattern was inversely associated with BW (β=−67.6 g per 1-unit increase; P<0.001) and increased risk of SGA; (OR=1.46; 95% CI 1.08 to 1.54; p=0.005).<br>-Not adjusted for cooking methods , among south-asians, maternal consumption of a plant-based diet was associated with a higher BW (β=+40.5 g per 1-unit increase; P=0.01).
Agnoli (2017)	[16]	Feasibility of vegetarian diets during pregnancy in Italy	Review	Average birthweights of infants born from vegetarian mothers do not differ significantly from the average of infants born from omnivorous mothers	Infants born to vegetarian mothers had low DHA in plasma and umbilical artery phospholipids
Gómez-Roig (2017)	[126]	To determine differences in lifestyle and dietary habits between pregnant women with SGA fetuses and those appropriate for gestational age AGA	Cross sectional N=127	Mothers of SGA fetuses had lower intake of micronutrients and cigarette smoking habits compared with mothers of AGA fetuses who ate more vegetables.	-Mothers maintaining a MD diet and lifestyle had an AGA fetus (P < 0.05).<br>-The same number of women in both groups ate a vegetarian diet.<br>-Women in the SGA group had a lower intake of: carotene, folic acid, iron, potassium and magnesium (P < 0.05).<br>-Women in the AGA group ate more

					vegetables, especially green beans, carrots, lettuce and oranges ($P < 0.05$). Bread, pasta, cakes and jam were also more frequently consumed by the AGA group ($P < 0.05$).<br>-Mothers in the SGA group drank more cola ($P < 0.05$), while mothers in the AGA group drank more diet cola and wine ($P < 0.05$).<br>-Women in the SGA group smoked more cigarettes per day ($P < 0.05$).
